# Inference for a Large Directed Acyclic Graph with Unspecified Interventions

**Published:** 2023

**Authors:** Chunlin Li, Xiaotong Shen, Wei Pan

**Affiliations:** School of Statistics, University of Minnesota, Minneapolis, MN 55455, USA; School of Statistics, University of Minnesota, Minneapolis, MN 55455, USA; Division of Biostatistics, University of Minnesota, Minneapolis, MN 55455, USA

**Keywords:** high-dimensional inference, data perturbation, structure learning, peeling algorithm, identifiability

## Abstract

Statistical inference of directed relations given some unspecified interventions (i.e., the intervention targets are unknown) is challenging. In this article, we test hypothesized directed relations with unspecified interventions. First, we derive conditions to yield an identifiable model. Unlike classical inference, testing directed relations requires to identify the ancestors and relevant interventions of hypothesis-specific primary variables. To this end, we propose a peeling algorithm based on nodewise regressions to establish a topological order of primary variables. Moreover, we prove that the peeling algorithm yields a consistent estimator in low-order polynomial time. Second, we propose a likelihood ratio test integrated with a data perturbation scheme to account for the uncertainty of identifying ancestors and interventions. Also, we show that the distribution of a data perturbation test statistic converges to the target distribution. Numerical examples demonstrate the utility and effectiveness of the proposed methods, including an application to infer gene regulatory networks. The R implementation is available at https://github.com/chunlinli/intdag.

## 1. Introduction

Directed relations are essential to explaining pairwise dependencies among multiple interacting units. In gene network analysis, regulatory gene-to-gene relations are a focus of biological investigation (Sachs et al., 2005), while in a human brain network, scientists investigate causal influences among regions of interest to understand how the brain functions through the regional effects of neurons (Liu et al., 2017). In such a situation, a Gaussian directed acyclic graph (DAG) is commonly employed to describe the directed relations; however, inferring the directed effects without other information is generally impossible because a Gaussian DAG often lacks model identifiability (Van de Geer and Bühlmann, 2013). Hence, external interventions are introduced to treat a non-identifiable situation (Heinze-Deml et al., 2018). For instance, the genetic variants such as single-nucleotide polymorphisms (SNPs) can be, and indeed are increasingly, treated as external interventions to infer inter-trait causal relations in a quantitative trait network (Brown and Knowles, 2020) and gene interactions in a gene regulatory network (Teumer, 2018; Molstad et al., 2021). In neuroimaging analysis, scientists use randomized experimental stimuli as interventions to identify causal relations in a functional brain network (Grosse-Wentrup et al., 2016; Bergmann and Hartwigsen, 2021). However, the interventions in these studies often have unknown targets and off-target effects (Jackson et al., 2003; Eaton and Murphy, 2007). Consequently, inferring directed relations while identifying useful interventions for inference is critical. This paper focuses on the simultaneous inference of directed relations subject to unspecified interventions (i.e., the intervention targets are unknown).

In a DAG model, the research has been centered on the reconstruction of directed relations in observational and interventional studies (Van de Geer and Bühlmann, 2013; Oates et al., 2016; Zheng et al., 2018; Yuan et al., 2019); see Heinze-Deml et al. (2018) for a review. For inference, Bayesian methods (Friedman and Koller, 2003; Luo and Zhao, 2011; Viinikka et al., 2020) have been popular. Yet, statistical inference remains under-studied, especially for interventional models in high dimensions (Peters et al., 2016; Rothenhäusler et al., 2019). Recently, for observational data, Janková and van de Geer (2018) propose a debiased test of the strength of a single directed relation, and Li et al. (2020) derives a constrained likelihood ratio test for multiple directed relations.

Despite progress, challenges remain. First, inferring directed relations requires to identify a certain DAG topological order (Van de Geer and Bühlmann, 2013), while the identifiability in a Gaussian DAG with unspecified interventions remains under-explored. Second, the inferential results should agree with the acyclicity requirement for a DAG. As a result, degenerate and intractable situations can occur, making inference greatly different from the classical ones. Third, likelihood-based methods for learning the DAG topological order often use permutation search (Van de Geer and Bühlmann, 2013) or continuous optimization subject to the acyclicity constraint (Zheng et al., 2018; Yuan et al., 2019), where a theoretical guarantee of the actual estimate (instead of the global optimum) has not been established for these approaches. Recently, an important line of work (Ghoshal and Honorio, 2018; Rajendran et al., 2021; Rolland et al., 2022) has focused on order-based algorithms with computational and statistical guarantees. However, in Gaussian DAGs, existing methods often rely on some error scale assumptions (Peters and Bühlmann, 2014), which is sensitive to variable scaling like the common practice of standardizing variables. This drawback could limit their applications, especially in causal inference, as causal relations are typically invariant to scaling.

To address the above issues, we develop structure learning and inference methods for a Gaussian DAG with unspecified additive interventions. Unlike the existing approach treating structure discovery and subsequent inference separately, our proposal integrates DAG structure learning and testing of directed relations, accounting for the uncertainty of structure learning for inference. With suitable interventions called instrumental variables (IVs), the proposed approach removes the restrictive error scale assumptions and delivers creditable outcomes with theoretical guarantees in low-order polynomial time. This indicates IVs, a well-known tool in causal inference (Angrist et al., 1996), can play important roles in structure learning even if some interventions do not meet the IV criteria. Our contributions are summarized as follows.

- For modeling, we establish the identifiability conditions for a Gaussian DAG with unspecified interventions. In particular, the conditions allow interventions on more than one target, which is suitable for multivariate causal analysis (Murray, 2006).
- For methodology, we develop likelihood ratio tests for directed edges and pathways in a super-graph of the true DAG, called the ancestral relation graph (ARG), where the ARG is formed by ancestral relations and candidate interventional relations, offering the topological order for inference. We reconstruct the ARG by the peeling algorithm, which automatically meets the acyclicity requirement. On this basis, we introduce the concepts of nondegeneracy and regularity to characterize the behavior of hypothesis testing under a DAG model. By integrating structure learning with inference, we account for the uncertainty of ARG estimation for the proposed tests via a novel data perturbation (DP) scheme, which effectively controls the type-I error while enjoying high statistical power.
- For theory, we prove that the proposed peeling algorithm based on nodewise regressions yields consistent results in $O\left(p\times \log\kappa_{\max}^{\circ }\times \left(q^{3}+nq^{2}\right)\right)$ operations almost surely, where *p*, *q* are the numbers of primary and intervention variables, *n* is the sample size, and $\kappa_{\max}^{\circ }$ is the sparsity. Then we justify the proposed DP inference method by establishing the convergence of the DP likelihood ratio to the target distribution and desired power properties.
- The numerical studies and real data analysis demonstrate the utility and effectiveness of the proposed methods. The implementation of the proposed tests and structure learning method is available at https://github.com/chunlinli/intdag.

The rest of the article is structured as follows. Section 2 establishes model identifiability and states two inference problems of interest. Section 3 develops the proposed methods for structure learning and statistical inference. Section 4 presents statistical theory to justify the proposed methods. Section 5 performs simulation studies, followed by an application to infer gene pathways from gene expression and SNP data in Section 6. Section 7 concludes the article. The Appendix contains illustrative examples and technical proofs.

## 2. Gaussian directed acyclic graph with additive interventions

To infer directed relations among *p* primary variables (i.e., variables of primary interest) $Y={\left(Y_{1},\ldots ,Y_{p}\right)}^{⊤}$, consider a structural equation model with *q* additive interventions:

(1) $$Y=U^{⊤}Y+W^{⊤}X+\varepsilon ,\;\varepsilon ∼N(0,\Sigma ),\;\Sigma =\text{Diag}\left(\sigma_{1}^{2},\ldots ,\sigma_{p}^{2}\right),$$

where $X={\left(X_{1},\ldots ,X_{q}\right)}^{⊤}$ is a vector of additive intervention variables, $U\in {\mathbb{R}}^{p\times p}$ and $W\in {\mathbb{R}}^{q\times p}$ are unknown coefficient matrices, and $\varepsilon ={\left(\varepsilon_{1},\ldots ,\varepsilon_{p}\right)}^{⊤}$ is a vector of random errors with $\sigma_{j}^{2}>0;j=1,\ldots ,p$. In (1), *ε* is independent of ***X*** but the components of ***X*** can be dependent. The matrix **U** specifies the directed relations among ***Y***, where ${\text{U}}_{kj}\neq 0$ if *Y_k_* is a direct cause of *Y_j_*, denoted by *Y_k_* → *Y_j_*, and *Y_k_* is called a parent of *Y_j_* or *Y_j_* a child of *Y_k_*. Thus, **U** represents a directed graph, which is further required to be acyclic to ensure the validity of the local Markov property (Spirtes et al., 2000). The matrix **W** specifies the targets and strengths of interventions, where ${\text{W}}_{lj}\neq 0$ indicates *X_l_* intervenes on *Y_j_*, denoted by *X_l_* → *Y_j_*. In (1), no directed edge from a primary variable *Y_j_* to an intervention variable *X_l_* is permissible.

In what follows, we will focus on the DAG 𝒢=(Y,X;ℰ,ℐ) with primary variables ***Y***, intervention variables ***X***, primary edges $ℰ=\left\{(k,j):{\text{U}}_{kj}\neq 0\right\}$, and intervention edges $ℐ=\left\{(l,j):{\text{W}}_{lj}\neq 0\right\}$. To facilitate discussion, we introduce some concepts and notations for 𝒢. If there is a directed path *Y_k_* → ⋯ → *Y_j_* in 𝒢, *Y_k_* is an ancestor of *Y_j_* or *Y_j_* is a descendant of *Y_k_*. If *Y_k_* → *Y_j_* and there is no other directed path from *Y_k_* to *Y_j_*, then we say *Y_k_* is an unmediated parent of *Y_j_*. Let ${\text{PA}}_{𝒢}(j)=\left\{k:Y_{k}\to Y_{j}\right\},{\text{AN}}_{𝒢}(j)=\left\{k:Y_{k}\to \cdots \to Y_{j}\right\}$, and ${\mathrm{IN}}_{G}(j)=\left\{l:X_{l}\to Y_{j}\right\}$ be the parent, ancestor, and intervention sets of *Y_j_* in DAG 𝒢, respectively.

### 2.1 Identifiability and instruments

Model (1) is generally non-identifiable without interventions (**W** = **0**) when errors do not meet some requirements such as the equal-variance assumption (Peters and Bühlmann, 2014) and its variants (Ghoshal and Honorio, 2018; Rajendran et al., 2021). Moreover, the model can be identified when *ε* in (1) is replaced by non-Gaussian errors (Shimizu et al., 2006) or linear relations are replaced by nonlinear ones (Peters et al., 2014). Regardless, suitable interventions can make (1) identifiable. When intervention targets are known, the identifiability issue has been studied (Oates et al., 2016; Chen et al., 2018). However, it is less so when the exact targets and strengths of interventions are unknown as in many biological applications (Jackson et al., 2003; Kulkarni et al., 2006), which is referred to as the case of *unspecified* or *uncertain* interventions (Heinze-Deml et al., 2018; Eaton and Murphy, 2007; Squires et al., 2020).

We now categorize interventions as instruments and invalid instruments.

**Definition 1 (DAG instrument)** *An intervention variable is an instrument in* 𝒢 *if*

- *it intervenes on at least one primary variable in* 𝒢;
- *it does not intervene on more than one primary variable in* 𝒢.

*Otherwise, it is an invalid instrument in* 𝒢.

Here, (A) requires an intervention to be active, while (B) prevents simultaneous interventions of a single intervention variable on multiple primary variables. This is critical to identifiability because an instrument on a (potential) cause variable *Y*_1_ helps reveal its directed effect on an outcome variable *Y*_2_, which breaks the symmetry in a Gaussian DAG that results in non-identifiability of directed relations *Y*_1_ → *Y*_2_ and *Y*_2_ → *Y*_1_.

**Remark 1** *In the literature* (Angrist et al., 1996), *an instrument X for estimating the effect from a potential cause Y*_1_ *to the outcome Y*_2_ *is required to satisfy (i) X is related to Y*_1_, *called relevance, (ii) X has no directed edge to the outcome Y*_2_, *called exclusion, and (iii) X is not related to unmeasured confounders, called unconfoundedness. In Definition 1, (A) is the relevance property, (B) generalizes the exclusion property for a DAG model, and the unconfoundedness is satisfied because no confounder is present in* model (1).

Next, we make some assumptions on intervention variables to yield an identifiable model, where dependencies among intervention variables are permissible.

**Assumption 1** *Assume that* model (1) *satisfies the following conditions*.

*(1A)* 𝔼 ***XX***^⊤^ *is positive definite*.
*(1B)* $\text{Cov}\left(Y_{j},X_{l}|X_{\left\{1,\ldots ,q\}\setminus \{l\right\}}\right)\neq 0$ *if X_l_ intervenes on any unmediated parent of Y_j_*.
*(1C) Each primary variable is intervened by at least one instrument*.

Assumption 1A imposes mild distributional restrictions on ***X***, permitting discrete variables such as SNPs. Assumption 1B requires the interventional effects through unmediated parents not to cancel out, as multiple targets from an invalid instrument are permitted. Importantly, if either Assumption 1B or 1C fails, model (1) is generally not identifiable, as shown in Example 2 of Appendix A.1. In Section 5, we empirically examine the situation when Assumption 1C is not met.

**Proposition 1** *Under* Assumption 1, (**U, W, Σ**) *in* model (1) *are identifiable from the distribution of* (**Y, X**).

Proposition 1 (proved in Appendix B.1) is derived for a DAG model with unspecified interventions. This is in contrast to Proposition 1 of Chen et al. (2018), which proves the identifiability of the parameters in a directed graph with target-known instruments on each primary variable. Moreover, it is worth noting that the estimated graph in Chen et al. (2018) may be cyclic and lacks the local Markov property for causal interpretation (Spirtes et al., 2000).

### 2.2 Problem statement: inference for a DAG

Our goal is to perform statistical inference of directed edges and pathways in the DAG 𝒢. Let ℋ⊆{(k,j):k≠j,1≤k,j≤p} be an edge set among primary variables {*Y*_1_, … , *Y_p_*}, where (k,j)∈ℋ specifies a (hypothesized) directed edge *Y_k_* → *Y_j_* in (1). We shall focus on two types of testing with null and alternative hypotheses *H*_0_ and *H_a_*. For simultaneous testing of directed edges,

(2) $$H_{0}:{\text{U}}_{kj}=0;\text{for all}\;(k,j)\in ℋ\;\text{versus}\;H_{a}:{\text{U}}_{kj}\neq 0\;\text{for some}\;(k,j)\in ℋ;$$

for simultaneous testing of directed pathways,

(3) $$H_{0}:{\text{U}}_{kj}=0;\text{for some}\;(k,j)\in ℋ\;\text{versus}\;H_{a}:{\text{U}}_{kj}\neq 0\;\text{for all}\;(k,j)\in ℋ,$$

where $\left(U_{{ℋ}^{c}},W,\Sigma \right)$ are unspecified nuisance parameters and *^c^* is the set complement. Note that *H*_0_ in (3) is a composite hypothesis that can be decomposed into sub-hypotheses

$$H_{0,\nu }:{\text{U}}_{k_{\nu },j_{\nu }}=0,\;\text{versus}\;H_{a,\nu }:{\text{U}}_{k_{\nu },j_{\nu }}\neq 0;\;\nu =1,\ldots ,|ℋ|,$$

where $ℋ=\left\{\left(k_{1},j_{1}\right),\ldots ,\left(k_{\left|ℋ\right|},j_{\left|ℋ\right|}\right)\right\}$ and testing each sub-hypothesis is a directed edge test. Thus, we treat (3) as an extension of (2).

We will also estimate (**U, W**) as well as identify the nonzero elements in **U** to recover the directed edges among the primary variables ***Y*** in 𝒢.

## 3. Methodology

This section develops the main methodology, including the peeling algorithm for structure learning and the data perturbation inference for simultaneous testing of directed edges (2) and pathways (3). To proceed, suppose the data matrices $Y_{n\times p}={\left(Y_{1},\ldots ,Y_{n}\right)}^{⊤}$ and $X_{n\times q}={\left(X_{1},\ldots ,X_{n}\right)}^{⊤}$ are given, where the rows ${\left\{\left(Y_{i}^{⊤},X_{i}^{⊤}\right)\right\}}_{1\leq i\leq n}$ are independently sampled from (1). Then the log-likelihood is (up to a constant)

(4) $$L(\theta ,\Sigma )=-\frac{1}{2}\overset{n}{\underset{i=1}{\sum }}{\left\|\Sigma^{-1/2}\left(\left(I-U^{⊤}\right)Y_{i}-W^{⊤}X_{i}\right)\right\|}_{2}^{2}-n\log\sqrt{\det(\Sigma )},$$

where $\theta =(U,W),\Sigma =\text{Diag}\left(\sigma_{1}^{2},\ldots ,\sigma_{p}^{2}\right)$, and **U** is subject to the acyclicity constraint (Zheng et al., 2018; Yuan et al., 2019) in that no directed cycle is permissible in the DAG.

One major challenge to this likelihood approach lies in the optimization of (4) subject to the acyclicity constraint, which imposes difficulty on not only computation but also asymptotic theory. As a result, there is a gap between the asymptotic distribution of a global maximum and that of the actual estimate which can be a local maximum (Janková and van de Geer, 2018; Li et al., 2020). Moreover, the actual estimate may give an imprecise topological order, tending to impact adversely on inference.

To circumvent the acyclicity requirement, we propose to use the ancestral relation graph (ARG) to describe the topological order of the DAG, where the ARG can be efficiently estimated without explicitly imposing the acyclicity constraint while enjoying a statistical guarantee of the actual estimate.

Definition 2 (Ancestral relation graph)

- *A graph* ℳ=(Y,X;𝒜,𝒞) *is an ARG if it is acyclic and* $𝒜=\left\{\left(k,j):k\in {\mathrm{AN}}_{ℳ}(j\right)\right\}$.
- *Given DAG* 𝒢=(Y,X;ℰ,ℐ), *its ARG is defined as* ${𝒢}_{+}=\left(Y,X;{ℰ}_{+},{ℐ}_{+}\right)$, *where*
$${ℰ}_{+}=\left\{\left(k,j):k\in {\text{AN}}_{𝒢}(j\right)\right\},\;{ℐ}_{+}=\left\{\left(l,j):l\in \underset{k\in {\text{AN}}_{𝒢}(j)\cup \{j\}}{⋃}{\text{IN}}_{𝒢}(k\right)\right\}.$$

Given ${𝒢}_{+}$ (which is acyclic), we have $\theta =\left(\theta_{{𝒢}_{+}},\theta_{{𝒢}_{+}^{c}}\right)$, where $\theta_{{𝒢}_{+}}=\left(U_{{ℰ}_{+}},W_{{ℐ}_{+}}\right)$ are the (effective) parameters and $\theta_{{𝒢}_{+}^{c}}=\left(U_{{ℰ}_{+}^{c}},W_{{ℐ}_{+}^{c}}\right)=0$. Then the log-likelihood (4) becomes

(5) $$L\left(\left(\theta_{{𝒢}_{+}},0\right),\Sigma \right)=-\overset{p}{\underset{j=1}{\sum }}\underset{:={\mathrm{RSS}}_{j}(\theta )}{\underset{︸}{\overset{n}{\underset{i=1}{\sum }}{\left(Y_{ij}-\underset{(k,j)\in {ℰ}_{+}}{\sum }U_{kj}Y_{ik}-\underset{(l,j)\in {ℐ}_{+}}{\sum }W_{lj}X_{il}\right)}^{2}}}/2\sigma_{j}^{2}+n\;\log\left(\sigma_{j}\right),$$

which involves $\left|{ℰ}_{+}\right|+\left|{ℐ}_{+}\right|$ parameters for $\theta_{{𝒢}_{+}}$. From (5), we can reconstruct 𝒢 and conduct inference for (2) and (3).

Our plan is as follows. In Section 3.1, we construct ${𝒢}_{+}$ without the acyclicity constraint for **U**. On this basis, in Section 3.2 we develop likelihood ratio tests for (2) and (3).

### 3.1 Structure learning via peeling

This section develops a novel structure learning method to construct ${𝒢}_{+}$ in a hierarchical manner. First, we observe an important connection between primary variables and intervention variables. Rewrite (1) as

(6) $$Y=V^{⊤}X+\varepsilon_{V},\;\varepsilon_{V}={\left(I-U^{⊤}\right)}^{-1}\varepsilon ∼N\left(0,\Omega^{-1}\right),$$

where $\Omega =(I-U)\Sigma^{-1}\left(I-U^{⊤}\right)$ and $V=W{\left(I-U\right)}^{-1}$.

**Proposition 2** *Suppose Assumption 1 is satisfied*.

- *If* ${\text{V}}_{lj}\neq 0$, *then X_l_ intervenes on Y_j_ or an ancestor of **Y**_j_;*
- *In* 𝒢, *Y_j_ is a leaf variable (having no child) if and only if there is an instrument X_l_ such that* ${\text{V}}_{lj}\neq 0$ *and* ${\text{V}}_{lj^{\prime }}=0$ *for* $j^{\prime }\neq j$.

The proof of Proposition 2 is deferred to Appendix B.2. Intuitively, ${\text{V}}_{lj}\neq 0$ implies the dependence of *Y_j_* on *X_l_* through a directed path *X_l_* → ⋯ → *Y_j_*, and hence that *X_l_* intervenes on *Y_j_* or an ancestor of *Y_j_*. Thus, the instruments on a leaf variable are independent of the other primary variables conditional on the rest of interventions. This observation suggests a method to reconstruct the DAG topological order by recursively identifying and removing (i.e., peeling) the leaf variables.

Next, we discuss the estimation of **V** and construction of ${𝒢}_{+}$.

#### 3.1.1 Nodewise constrained regressions

We estimate **V** = (**V**_·1_, … , **V**_·*p*_) via nodewise ℓ_0_-constrained regressions,

(7) $${\hat{V}}_{\cdot j}=\underset{V_{\cdot j}}{\mathrm{arg min}}\overset{n}{\underset{i=1}{\sum }}{\left(Y_{ij}-V_{\cdot j}^{⊤}X_{i}\right)}^{2}\;\text{s.t.}\;\overset{q}{\underset{l=1}{\sum }}\text{I}\left({\text{V}}_{lj}\neq 0\right)\leq \kappa_{j};\;j=1,\ldots ,p,$$

where $1\leq \kappa_{j}\leq q$ is an integer-valued tuning parameter controlling the sparsity and can be chosen by BIC or cross-validation. To solve (7), we use $J\left(z;\tau_{j}\right)=\min\left(|z|/\tau_{j},1\right)$ as a surrogate of **I**(*z* ≠ 0) (Shen et al., 2012) and develop a difference-of-convex (DC) program with the ℓ_0_-projection to improve the globality of the solution of (7). Specifically, at the (*t* + 1)th iteration, given ${\tilde{V}}_{\cdot j}^{\left[t\right]}$, we solve the weighted Lasso problem,

(8) $${\tilde{V}}_{\cdot j}^{\left[t+1\right]}=\underset{V_{\cdot j}}{\mathrm{arg min}}\overset{n}{\underset{i=1}{\sum }}{\left(Y_{ij}-V_{\cdot j}^{⊤}X_{i}\right)}^{2}+2n\gamma_{j}\tau_{j}\overset{q}{\underset{l=1}{\sum }}\text{I}\left(\left|{\tilde{\text{V}}}_{lj}^{\left[t\right]}\right|\leq \tau_{j}\right)\left|{\text{V}}_{lj}\right|;\;j=1,\ldots ,p,$$

where $\gamma_{j}>0$ is an internal hyperparameter used by the DC program; see Remark 2 below. The DC program terminates at ${\tilde{V}}_{\cdot j}={\tilde{V}}_{\cdot j}^{\left[t\right]}$ such that ${\left\|{\tilde{V}}_{\cdot j}^{\left[t+1\right]}-{\tilde{V}}_{\cdot j}^{\left[t\right]}\right\|}_{\infty }\leq \sqrt{\text{tol}}$ or *t* achieves the maximum iteration number, where tol is the machine precision. Then, the solution ${\hat{V}}_{\cdot j}$ of (7) is computed by projecting ${\tilde{V}}_{\cdot j}$ onto the set $\left\{v\in {\mathbb{R}}^{q}:∥v{∥}_{0}\leq \kappa_{j}\right\}$.

Algorithm 1 summarizes the computation method.


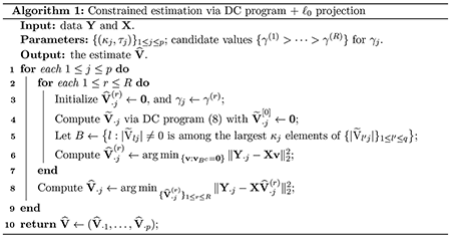


**Remark 2** *In* Algorithm 1, *γ_j_ is chosen from a set of candidate values* ${\left\{\gamma^{\left(r\right)}\right\}}_{1\leq r\leq R}$. *In our implementation, γ_j_ is not directly tuned by the user and* ${\left\{\gamma^{\left(r\right)}\right\}}_{1\leq r\leq R}$ *is provided by default. When* ${\left\{\left(\kappa_{j},\tau_{j}\right)\right\}}_{1\leq j\leq p}$ *are suitably specified by the user, for any value γ*^(*r*)^ *lies in the proper ranges*, $\left(V_{\cdot 1},\ldots ,V_{p}\right)$ *are the global solutions of* (7) *almost surely; see Theorem 4 in* Section 4.1. *Moreover, solving the DC programs for* $\gamma^{\left(1\right)}>\cdots >\gamma^{\left(R\right)}$ *is efficient with the warm start trick* (Friedman et al., 2010; Breheny and Huang, 2011).

#### 3.1.2 Peeling

Now, we describe a *peeling* algorithm to estimate ${𝒢}_{+}$ based on **V**. Proposition 2 suggests that the leaf variables of 𝒢 (with their instruments) can be identified based on matrix **V**. To proceed, let ℒ and its complement ${ℒ}^{c}$ be (generic) nonempty subsets of {1, … , *p*} such that $Y_{{ℒ}^{c}}$ are non-descendants of $Y_{ℒ}$. Define a sub-DAG ${𝒢}_{{ℒ}^{c}}=\left(Y_{{ℒ}^{c}},X;{ℰ}_{{ℒ}^{c}},{ℐ}_{{ℒ}^{c}}\right)$, where ${ℰ}_{{ℒ}^{c}}\subseteq ℰ$ is the set of primary edges among $Y_{{ℒ}^{c}}$ and ${ℐ}_{{ℒ}^{c}}\subseteq ℐ$ is the set of intervention edges between ***X*** and $Y_{{ℒ}^{c}}$. The following proposition offers insights into the connection between **V** and ${𝒢}_{+}$.

**Proposition 3** *Suppose* Assumption 1 *is satisfied*. *Let Y_k_ be a leaf in* ${𝒢}_{{ℒ}^{c}}$ *and Y_j_ be in* $Y_{{ℒ}^{c}}$.

- *If* ${\text{V}}_{lj}\neq 0$ *for each instrument X_l_ of Y_k_ in* ${𝒢}_{{ℒ}^{c}}$, *we have* $(k,j)\in {ℰ}_{+}$.
- *If Y_k_ is an unmediated pa/rent of Y_j_, then* ${\text{V}}_{lj}\neq 0$ *for each instrument X_l_ of Y_k_ in* ${𝒢}_{{ℒ}^{c}}$.

Proposition 3 (proved in Appendix B.3) together with Proposition 2 indicates that ${𝒢}_{+}$ can be constructed from **V**. In particular, we can sequentially identify each leaf *Y_k_* with its instrument(s) *X_l_* in the DAG 𝒢 or the sub-DAG ${𝒢}_{{ℒ}^{c}}$ (where $Y_{ℒ}$ are peeled variables). Then ${ℰ}_{+}$ can be constructed by including all edges (*k, j*) such that *Y_k_* is a leaf in the sub-DAG ${𝒢}_{{ℒ}^{c}}$, *Y_j_* is a peeled variable, and ${\text{V}}_{lj}\neq 0$ for each instrument *X_l_* of leaf *Y_k_* in ${𝒢}_{{ℒ}^{c}}$. By Proposition 3, (A) confirms that all such edges are in ${ℰ}_{+}$ so no extra edges are included, and (B) guarantees that every directed edge from an unmediated parent must be included, which is sufficient to determine all the ancestral relations. Thus, ${ℰ}_{+}$ can be recovered from **V**. Then ${ℐ}_{+}$ is equal to $\left\{(l,j):{\text{V}}_{lk}\neq 0\;\text{if}\;k=j\;\text{or}\;(k,j)\in {ℰ}_{+}\right\}$.

The peeling algorithm is summarized in Algorithm 2 and a detailed illustration is presented in Example 3 of Appendix A.2.


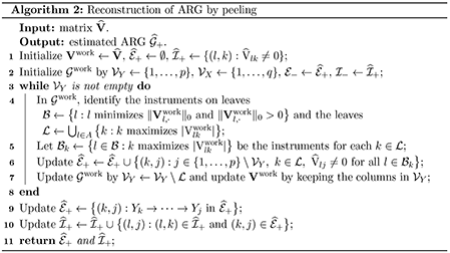


**Remark 3** *Given* ${\hat{𝒢}}_{+}=\left(Y,X;{\hat{ℰ}}_{+},{\hat{ℐ}}_{+}\right)$, *we estimate* $\left(U_{{\hat{ℰ}}_{+}},W_{{\hat{ℐ}}_{+}}\right)$ *column-wise from* (5),

(9) $$\min\overset{n}{\underset{i=1}{\sum }}{\left(Y_{ij}-\underset{k\in {\text{AN}}_{{\hat{𝒢}}_{+}}(j)}{\sum }{\text{U}}_{kj}Y_{ik}-\underset{l\in {\text{IN}}_{{\hat{𝒢}}_{+}}(j)}{\sum }{\text{W}}_{lj}X_{il}\right)}^{2}\text{s.t.}\underset{k\in {\text{AN}}_{{\hat{𝒢}}_{+}}(j)}{\sum }\text{I}\left({\text{U}}_{kj}\neq 0\right)\leq {\kappa^{\prime }}_{j}.$$

*The final estimates are* $\hat{\text{U}}=\left({\hat{\text{U}}}_{{\hat{ℰ}}_{+}},0\right)$ *and* $\hat{\text{W}}=\left({\hat{\text{W}}}_{{\hat{ℐ}}_{+}},0\right)$. *In* (9), *the sparsity constraint is imposed to recover the nonzero elements of* **U**; *see* Appendix A.6 *for the technical discussion*.

### 3.2 Likelihood inference for a DAG

Now, we propose an inference method for testing (2) and (3). First, we derive the likelihood ratio based on ${𝒢}_{+}$. Next, we perform tests via data perturbation, accounting for the uncertainty of estimating ${𝒢}_{+}$.

#### 3.2.1 Likelihood ratio, nondegeneracy, and regularity

We commence with the likelihood inference for (2), since (3) can be treated as an extension of (2); see the discussion followed by (3). From (5), the maximum likelihood becomes

$$\underset{{𝒢}_{+},\text{Σ}}{\max}\underset{\theta =\left(\theta_{{𝒢}_{+}},0\right)}{\max}L(\theta ,\Sigma ).$$

Thus, we define the likelihood ratio by

(10) $$\mathrm{Lr}=L\left({\hat{\theta }}^{\left(1\right)},\hat{\Sigma }\right)-L\left({\hat{\theta }}^{\left(0\right)},\hat{\Sigma }\right)=\overset{p}{\underset{j=1}{\sum }}\left({\text{RSS}}_{j}\left({\hat{\theta }}^{\left(0\right)}\right)-{\text{RSS}}_{j}\left({\hat{\theta }}^{\left(1\right)}\right)\right)/2{\hat{\sigma }}_{j}^{2},$$

where ${\hat{\theta }}^{\left(0\right)}=\left({\hat{\theta }}_{\hat{ℳ}}^{\left(0\right)},0\right)$ and ${\hat{\theta }}^{\left(1\right)}=\left({\hat{\theta }}_{\hat{ℳ}}^{\left(1\right)},0\right)$ are MLEs (given $\hat{ℳ}$) under *H*_0_ and *H_a_*, respectively, $\hat{ℳ}=\left(Y,X;{\hat{ℰ}}_{+}\cup ℋ,{\hat{ℐ}}_{+}\right)$ is an estimate for ${𝒢}_{+}$, and $\hat{\text{Σ}}$ is an estimate for $\hat{\text{Σ}}$. Instead of ${\hat{𝒢}}_{+}=\left(Y,X;{\hat{ℰ}}_{+},{\hat{ℐ}}_{+}\right)$, the graph $\hat{ℳ}$ (with hypothesized edges being added) is used because we need to test the presence of any edge in ℋ.

In many statistical models, the likelihood ratio often has a nondegenerate and tractable distribution when *H*_0_ is true, for instance, a chi-squared distribution with degrees of freedom |ℋ|. However, since ℋ is pre-specified by the user, $\hat{ℳ}$ may not be a DAG, and thus not all edges in ℋ could present in the DAG parameterized by $\left({\hat{\theta }}_{\hat{ℳ}}^{\left(1\right)},0\right)$. As a result, Lr for (2) may converge to a distribution with degrees of freedom less than |ℋ| and the distribution may be even intractable, making inference for a DAG greatly different from the classical ones, as illustrated by Example 1.

**Figure 1:**
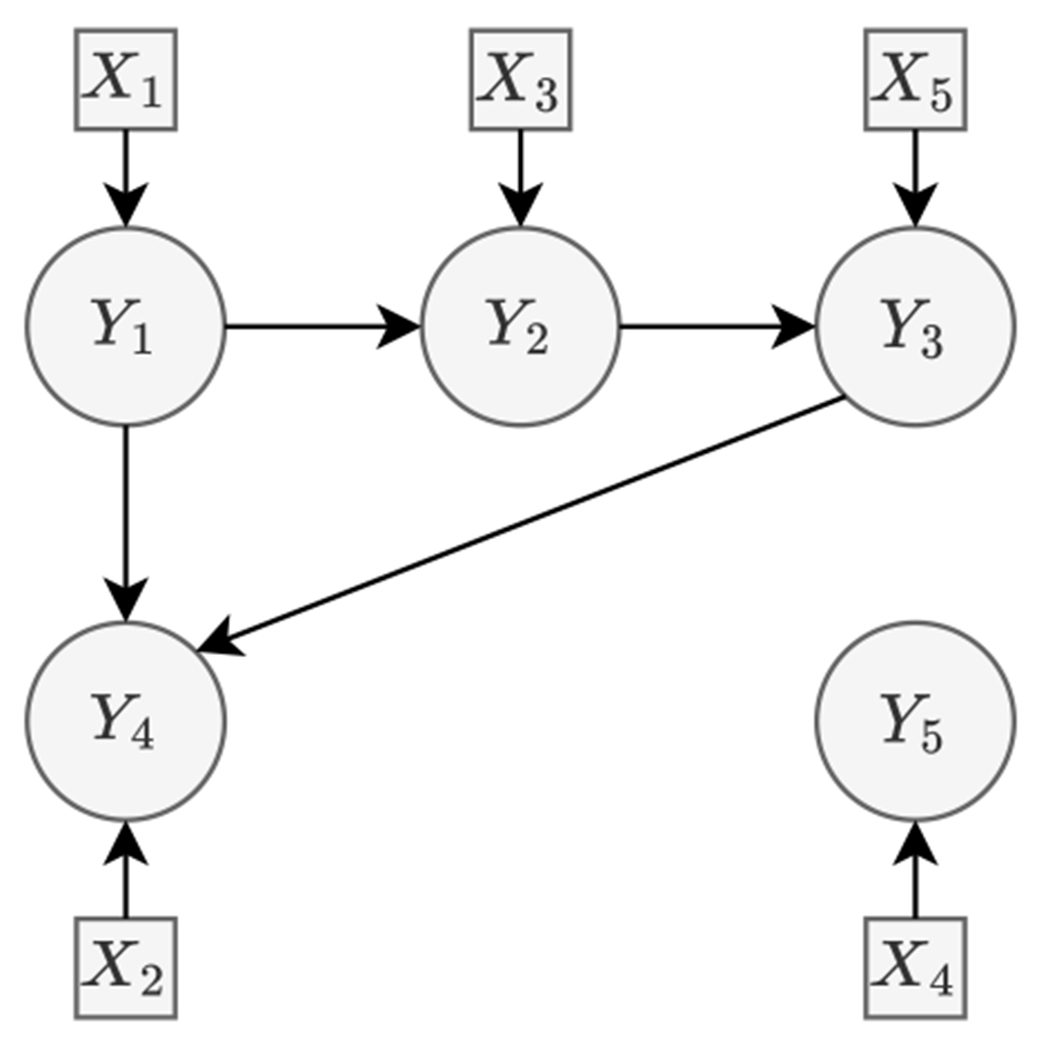
A DAG 𝒢 of five primary variables *Y*_1_, … , *Y*_5_ and five intervention variables *X*_1_, … , *X*_5_, where directed edges are represented by solid arrows while dependencies among ***X*** are not displayed.

**Example 1** *Consider the likelihood ratio test under null H*_0_ *and alternative H_a_ for the DAG* 𝒢 *displayed in* Figure 1. *For simplicity, assume* ${\hat{𝒢}}_{+}={𝒢}_{+}$ *and* $\hat{ℳ}=\left(Y,X;{ℰ}_{+}\cup ℋ,{ℐ}_{+}\right)$.

- *H*_0_ : *U*_21_ = 0 *versus* $H_{a}:{\text{U}}_{21}\neq 0$, *where* ℋ={(2,1)}. *Here*, (2, 1) *forms a cycle together with the edges in* ℰ∖ℋ *(namely the edges not considered by the hypothesis), and thus* $\hat{ℳ}$ *has a directed cycle. Given* $\hat{ℳ}$, *when a random sample is obtained under H*_0_, *the likelihood tends to be maximized under the ARG* ${𝒢}_{+}$ *corresponding to the underlying DAG (which implies* U_21_ = 0), *especially so when the asymptotics kicks in as the sample size increases. Consequently, the likelihood ratio Lr becomes zero, constituting a degenerate situation*.
- *H*_0_ : U_45_ = U_53_ = 0 *versus* $H_{a}:{\text{U}}_{45}\neq 0$ *or* ${\text{U}}_{53}\neq 0$, *where* ℋ={(4,5),(5,3)}. *In this case*, {(4, 5), (5, 3)} *forms a cycle with the edges in* ℰ∖ℋ, *and* $\hat{ℳ}$ *is cyclic. Given* $\hat{ℳ}$, *the likelihood tends to be maximized under either* $DAG\;\left(Y,X;{ℰ}_{+}\cup \{(4,5)\},{ℐ}_{+}\right)$ *or* $DAG\;\left(Y,X;{ℰ}_{+}\cup \{(5,3)\},{ℐ}_{+}\right)$ *when data is sampled under H*_0_. *Thus, we have*
$$L\left({\hat{\theta }}^{\left(1\right)},\hat{\Sigma }\right)=\max\left(L\left({\hat{\text{U}}}_{45},{\hat{\text{U}}}_{53}=0,{\hat{\text{U}}}_{{ℋ}^{c}},\hat{W},\hat{\Sigma }\right),L\left({\hat{\text{U}}}_{45}=0,{\hat{\text{U}}}_{53},{\hat{\text{U}}}_{{ℋ}^{c}},\hat{W},\hat{\Sigma }\right)\right).$$

*As a result, the likelihood ratio distribution becomes complicated in this situation due to the dependence between the two components in* $L\left({\hat{\theta }}^{\left(1\right)},\hat{\Sigma }\right)$.

Motivated by Example 1, we introduce the concepts of nondegeneracy and regularity.

Definition 3 (Nondegeneracy and regularity with respect to DAG)

- *An edge* (k,j)∈ℋ *is nondegenerate with respect to DAG* 𝒢 *if* {(*k, j*)} ∪ ℰ *contains no directed cycle, where* ℰ *denotes the edge set of* 𝒢. *Otherwise*, (*k, j*) *is degenerate. Let* 𝒟⊆ℋ *be the set of all nondegenerate edges with respect to* 𝒢. *A null hypothesis H*_0_ *is nondegenerate with respect to DAG* 𝒢 *if* 𝒟≠∅. *Otherwise, H*_0_ *is degenerate*.
- *A null hypothesis H*_0_ *is said to be regular with respect to DAG* 𝒢 *if* 𝒟∪ℰ *contains no directed cycle, where* ℰ *denotes the edge set of* 𝒢. *Otherwise, H*_0_ *is called irregular*.

**Remark 4** *In practice*, 𝒟 *is unknown and needs to be estimated from data. Indeed*, $\hat{𝒟}$ *can be computed based on the estimated ARG* ${\hat{𝒢}}_{+}$, *because a directed edge* (*k, j*) *is nondegenerate if and only if* {(*k, j*)} ∪ ℰ_+_ *contains no directed cycle*.

Nondegeneracy ensures nonnegativity of the likelihood ratio. In testing (2), regularity excludes intractable situations for the null distribution. In testing (3), if *H*_0_ is irregular, then 𝒟∪ℰ has a directed cycle, which means the hypothesized directed pathway cannot exist due to the acyclicity constraint. Thus, regularity excludes the degenerate situations in testing (3). In what follows, we mainly focus on nondegenerate and regular hypotheses. For the degenerate case, the p-value is defined to be one. For the irregular case of edge test (2), we decompose the hypothesis into regular sub-hypotheses and conduct multiple testing. For the irregular case of pathway test (3), the p-value is defined to be one. More discussions on the implementation in irregular cases are provided in Appendix A.5.

#### 3.2.2 Testing directed edges via data perturbation

Assuming *H*_0_ is nondegenerate and regular, then ${\hat{\theta }}^{\left(1\right)}$ is the MLE subject to the DAG $\hat{𝒮}=\left(Y,X;{\hat{ℰ}}_{+}\cup \hat{𝒟},{\hat{ℐ}}_{+}\right)$ and ${\hat{\theta }}^{\left(0\right)}$ is the MLE subject to an additional constraint ${\text{U}}_{ℋ}=0$. The likelihood ratio (10) can be further simplified. Let ${\text{D}}_{\hat{𝒮}}(j)=\{k:(k,j)\in \hat{𝒟}\}$ in DAG $\hat{𝒮}$, where $\hat{𝒟}$ is the estimated set of nondegenerate edges of *H*_0_ with respect to 𝒢. Furthermore, observe that if ${\text{D}}_{\hat{𝒮}}(j)=\emptyset$, then ${\text{RSS}}_{j}\left({\hat{\theta }}^{\left(0\right)}\right)={\text{RSS}}_{j}\left({\hat{\theta }}^{\left(1\right)}\right)$. Hence, Lr only summarizes the contributions from the primary variables with the (estimated) nondegenerate hypothesized edges,

(11) $$\text{Lr}=\sum_{\left\{j:{\text{D}}_{\hat{𝒮}}(j)\neq \emptyset \right\}}\left({\mathrm{RSS}}_{j}\left({\hat{\theta }}^{\left(0\right)}\right)-{\mathrm{RSS}}_{j}\left({\hat{\theta }}^{\left(1\right)}\right)\right)/2{\hat{\sigma }}_{j}^{2},$$

where we estimate $\text{Σ}=\text{Diag}\left(\sigma_{j}^{1},\ldots ,\sigma_{p}^{2}\right)$ by

(12) $${\hat{\sigma }}_{j}^{2}={\text{RSS}}_{j}\left({\hat{\theta }}^{\left(1\right)}\right)/\left(n-\left|{\text{PA}}_{\hat{𝒮}}(j)\right|-\left|{\text{IN}}_{\hat{𝒮}}(j)\right|\right),\;j=1,\ldots ,p.$$

The likelihood ratio (11) for testing directed edges (2) requires an estimation of ${𝒢}_{+}$ (and 𝒮), where we must account for the uncertainty of ${\hat{𝒢}}_{+}$ (and $\hat{𝒮}$) for finite-sample inference. To proceed, we consider the test statistic Lr based on a “correct” ARG $ℳ⊇{𝒢}_{+}$, where $ℳ=(Y,X;𝒜,𝒞)⊇G_{+}=\left(Y,X;{ℰ}_{+},{ℐ}_{+}\right)$ means that $𝒜⊇{ℰ}_{+}$ and $𝒞⊇{ℐ}_{+}$. Intuitively, a “correct” ARG distinguishes descendants and nondescendants, and thus can help infer the true directed relations defined by the local Markov property (Spirtes et al., 2000) without introducing model errors, yet may lead to a less powerful test when ℳ is much larger than ${𝒢}_{+}$. By comparison, a “wrong” ARG $ℳ⊉{𝒢}_{+}$ provides an incorrect topological order, and a test based on a “wrong” ARG may be biased, accompanied by an inflated type-I error.

Let **Z** = (**Y, X**) denote the data matrix of primary and intervention variables and let $e={\left(\varepsilon_{1},\ldots ,\varepsilon_{n}\right)}^{⊤}$ denote the error matrix, where the rows ${\left\{Z_{i,\cdot }=\left(Y_{i}^{⊤},X_{i}^{⊤}\right)\right\}}_{1\leq i\leq n}$ and ${\left\{\varepsilon_{i}^{⊤}\right\}}_{1\leq i\leq n}$ are sampled independently from (1). From (11), assuming ${\hat{𝒢}}_{+}⊇{𝒢}_{+}$ is a “correct” ARG, the likelihood ratio becomes

(13) $$\text{Lr}=\sum_{\left\{j:{\text{D}}_{\hat{𝒮}}(j)\neq \emptyset \right\}}\frac{{\left\|{\left(P_{{\hat{A}}_{j}}-P_{{\hat{B}}_{j}}\right)}^{1/2}Y_{\cdot j}\right\|}_{2}^{2}}{2{\left\|{\left(I-P_{{\hat{A}}_{j}}\right)}^{1/2}Y_{\cdot j}\right\|}_{2}^{2}/\left(n-\left|{\hat{A}}_{j}\right|\right)}\;\;=\underset{\left\{j:{\text{D}}_{\hat{𝒮}}(j)\neq \emptyset \right\}}{\sum }\frac{{\left\|{\left(P_{{\hat{A}}_{j}}-P_{{\hat{B}}_{j}}\right)}^{1/2}\left(Y_{\cdot ,{\text{D}}_{\hat{𝒮}}(j)}U_{{\text{D}}_{\hat{𝒮}}(j),j}+e_{\cdot j}\right)\right\|}_{2}^{2}}{2{\left\|{\left(I-P_{{\hat{A}}_{j}}\right)}^{1/2}e_{\cdot j}\right\|}_{2}^{2}/\left(n-\left|{\hat{A}}_{j}\right|\right)},$$

where $P_{A}=Z_{\cdot ,A}{\left(Z_{\cdot ,A}^{⊤}Z_{\cdot ,A}\right)}^{-1}Z_{\cdot ,A}^{⊤}$ is the projection matrix onto the column span of $Z_{\cdot ,A},{\hat{A}}_{j}={\mathrm{PA}}_{\hat{𝒮}}(j)\cup {\text{IN}}_{\hat{𝒮}}(j)$, and ${\hat{B}}_{j}=\left({\text{PA}}_{\hat{𝒮}}(j)\cup {\text{IN}}_{\hat{𝒮}}(j)\right)\setminus {\text{D}}_{\hat{𝒮}}(j)$ for 1≤j≤p. In (13), we have $Y_{\cdot ,{\text{D}}_{𝒮}(j)}U_{{\text{D}}_{𝒮}(j),j}=0$ for all *j* under the null hypothesis *H*_0_, while $Y_{{\text{D}}_{𝒮}(j)}U_{{\text{D}}_{𝒮}(j),j}\neq 0$ for some *j* under the alternative hypothesis *H_a_*, and thus Lr tends to be large under *H_a_*.

Now, we propose the data perturbation (DP) method (Shen and Ye, 2002; Breiman, 1992) to approximate the null distribution of Lr in (13). The idea behind DP is to assess the sensitivity of the estimates through perturbed data Y* = **Y** + e*, where the rows ${\left\{{\left(\varepsilon_{i}^{*}\right)}^{⊤}\right\}}_{1\leq i\leq n}$ of perturbation errors $e_{n\times p}^{*}$ is sampled independently from $N(0,\hat{\text{Σ}})$. Let (Z*, e*) = (**X**, Y*, e*) be the DP sample. Note that the perturbation errors e* are only injected into **Y** and the perturbation errors e* are known in the DP sample (Z*, e*). Given (Z*, e*), we compute the perturbation estimate ${\hat{𝒢}}_{+}^{*}$ (and ${\hat{𝒮}}^{*}$) by Algorithms 1–2. In (13), under the null hypothesis *H*_0_, the likelihood ratio Lr = Λ(**Z**, **e**) is a function of observed data **Z** and unobserved errors **e**. By definition, the perturbation error e* is accessible in the DP sample (Z*, e*), suggesting the DP likelihood ratio Lr* := Λ(Z*, e*) that is equal to

(14) $${\text{Lr}}^{*}=\underset{\left\{j:{\text{D}}_{\hat{S}}(j)\neq \emptyset \right\}}{\sum }\frac{{\left\|{\left(P_{{\hat{A}}_{j}^{*}}^{*}-P_{{\hat{B}}_{j}^{*}}^{*}\right)}^{1/2}e_{\cdot j}^{*}\right\|}_{2}^{2}}{2{\left\|{\left(I-P_{{\hat{A}}_{j}^{*}}^{*}\right)}^{1/2}e_{\cdot j}^{*}\right\|}_{2}^{2}/\left(n-\left|{\hat{A}}_{j}^{*}\right|\right)}.$$

Note that (14) mimics (13) when $Y_{\cdot ,{\text{D}}_{S}(j)}U_{{\text{D}}_{S}(j)}=0$. As a result, when ${\hat{𝒢}}_{+}^{*}⊇{𝒢}_{+}$, the conditional distribution of Lr* given the data **Z** well approximates the null distribution of Lr, where the model selection effect is accounted for by assessing the variability of ${\left\{{\hat{A}}_{j}^{*},{\hat{B}}_{j}^{*}\right\}}_{1\leq j\leq p}$ over different realizations of (Z*, e*).

In practice, we use Monte-Carlo to approximate the distribution of Lr* given **Z**. We generate *M* perturbed samples ${\left\{\left(Z_{m}^{*},e_{m}^{*}\right)\right\}}_{1\leq m\leq M}$ independently and compute ${\left\{{\text{Lr}}_{m}^{*}\right\}}_{1\leq m\leq M}$, respectively. Then, we examine the condition ${\hat{𝒢}}_{+,m}^{*}⊇{𝒢}_{+}$ by checking its empirical counterpart ${\hat{𝒢}}_{+,m}^{*}⊇{\hat{𝒢}}_{+}$. The DP p-value of the edge test in (2) is defined as

(15) $$\text{Pval}=\left(\overset{M}{\underset{m=1}{\sum }}\text{I}\left({\text{Lr}}_{m}^{*}\geq \text{Lr},{\hat{𝒢}}_{+,m}^{*}⊇{\hat{𝒢}}_{+}\right)\right)/\left(\overset{M}{\underset{m=1}{\sum }}\text{I}\left({\hat{𝒢}}_{+,m}^{*}⊇{\hat{𝒢}}_{+}\right)\right),$$

where **I**(·) is the indicator function.

**Remark 5** *Instead of* (14), *a naive approach is to recompute the likelihood ratio by treating the perturbed sample* Z* as **Z** *while not using the information of* e*. *However, this is infeasible. For explanation, assuming* ${\hat{𝒢}}_{+}^{*}⊇{𝒢}_{+}$ *is a “correct” ARG, then this naive likelihood ratio is equal to*

$$\underset{\left\{j:{\text{D}}_{{\hat{𝒮}}^{*}}(j)\neq \emptyset \right\}}{\sum }\frac{{\left\|{\left(P_{{\hat{A}}_{j}^{*}}^{*}-P_{{\hat{B}}_{j}^{*}}^{*}\right)}^{1/2}\left(Y_{\cdot ,j}+e_{\cdot j}^{*}\right)\right\|}_{2}^{2}}{2{\left\|{\left(I-P_{{\hat{A}}_{j}^{*}}^{*}\right)}^{1/2}\left(Y_{\cdot j}+e_{\cdot j}^{*}\right)\right\|}_{2}^{2}/\left(n-\left|{\hat{A}}_{j}^{*}\right|\right)}.$$

*Note that* ${\left\{Y_{\cdot ,j}\right\}}_{1\leq j\leq p}$ *given* **Z** *are deterministic and do not vanish under either H*_0_ *or H_a_. Thus, the conditional distribution of this naive likelihood ratio given* **Z** *does not approximate the null distribution of Lr, in contrast to the DP likelihood ratio in* (14).

#### 3.2.3 Extension to hypothesis testing for a directed pathway

Next, we extend the DP inference for (3). Denote $ℋ=\left\{\left(k_{1},j_{1}\right),\ldots ,\left(k_{\left|ℋ\right|},j_{\left|ℋ\right|}\right)\right\}$. Then the test of pathways in (3) can be reduced to testing sub-hypotheses

$$H_{0,\nu }:{\text{U}}_{k_{\nu },j_{\nu }}=0,\;\text{versus}\;H_{a,\nu }:{\text{U}}_{k_{\nu },j_{\nu }}\neq 0;\;\nu =1,\ldots ,|ℋ|,$$

where testing each sub-hypothesis is a directed edge test. Given $\left(\hat{𝒮},\hat{\text{Σ}}\right)$, the likelihood ratio for *H*_0_,*_ν_* is ${\text{Lr}}_{\nu }=L\left({\hat{\theta }}^{\left(1\right)},\hat{\Sigma }\right)-L\left({\hat{\theta }}^{\left(0,\nu \right)},\hat{\Sigma }\right)$, where ${\hat{\theta }}^{\left(0,\nu \right)}$ is the MLE under the constraint that ${\text{U}}_{k_{\nu },j_{\nu }}=0$. When ${\hat{𝒢}}_{+}⊇{𝒢}_{+}$, we have

(16) $${\text{Lr}}_{\nu }=\frac{{\left\|{\left(P_{{\hat{A}}_{j_{\nu }}}-P_{{\hat{B}}_{j_{\nu }}}\right)}^{1/2}\left(Y_{k_{\nu }}{\text{U}}_{k_{\nu },j_{\nu }}+e_{\cdot j_{\nu }}\right)\right\|}_{2}^{2}}{2{\left\|{\left(I-P_{{\hat{A}}_{j_{\nu }}}\right)}^{1/2}e_{\cdot j_{\nu }}\right\|}_{2}^{2}/\left(n-\left|{\hat{A}}_{j_{\nu }}\right|\right)},{\text{Lr}}_{\nu }^{*}=\frac{{\left\|{\left(P_{{\hat{A}}_{j_{\nu }}^{*}}^{*}-P_{{\hat{B}}_{j_{\nu }}^{*}}^{*}\right)}^{1/2}e_{\cdot j_{\nu }}^{*}\right\|}_{2}^{2}}{2{\left\|{\left(I-P_{{\hat{A}}_{j_{\nu }}^{*}}^{*}\right)}^{1/2}e_{\cdot j_{\nu }}^{*}\right\|}_{2}^{2}/\left(n-\left|{\hat{A}}_{j_{\nu }}^{*}\right|\right)},$$

where the distributions of ${\text{Lr}}_{\nu }^{*}$ given **Z** approximates the null distributions of Lr_*ν*_. Finally, define the p-value of the pathway test in (3) as

(17) $$\text{Pval}=\underset{1\leq \nu \leq |ℋ|}{\max}\left(\overset{M}{\underset{m=1}{\sum }}\text{I}\left({\text{Lr}}_{\nu ,m}^{*}\geq {\text{Lr}}_{\nu },{\hat{S}}_{m}^{*}⊇\hat{S}\right)\right)/\left(\overset{M}{\underset{i=1}{\sum }}\text{I}\left({\hat{S}}_{m}^{*}⊇\hat{S}\right)\right).$$

Note that if any *H*_0,*ν*_ is degenerate, then Pval = 1.

Algorithm 3 summarizes the DP method for hypothesis testing.


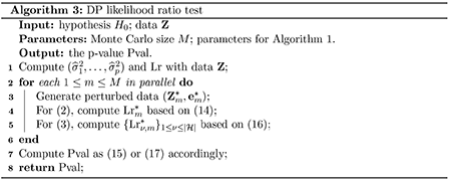


**Remark 6** *For acceleration, we parallelize Step 2 in Algorithm 3. Additionally, we use the estimate* $\hat{\theta }$ *as a warm-start initialization for the DP estimates, effectively reducing the computing time*.

**Remark 7 (Connection with bootstrap)** *One may consider parametric or nonparametric bootstrap for Lr. The parametric bootstrap requires a good initial estimate of* (**U, W**). *Yet, it is rather challenging to correct the bias of this estimate because of the acyclicity constraint. By comparison, DP does not rely on such an estimate. On the other hand, nonparametric bootstrap resamples the original data with replacement. In a bootstrap sample, only about 63% distinct observations in the original data are used in model selection and fitting, leading to deteriorating performance* (Kleiner et al., 2012), *especially in a small sample. As a result, nonparametric bootstrap may not well-approximate the distribution of Lr, while DP provides a better approximation of Lr, taking advantage of a full sample.*

## 4. Theory

This section provides the theoretical justification for the proposed methods.

### 4.1 Convergence and consistency of structure learning

First, we introduce some technical assumptions to derive statistical and computational properties of Algorithms 1 and 2. Let *ζ* be a generic vector and *ζ_A_* be the subvector of *ζ* with coordinates in *A*. Let $\kappa_{j}^{\circ }={\left\|V_{\cdot j}\right\|}_{0}$ and $\kappa_{\max}^{\circ }=\max_{1\leq j\leq p}\kappa_{j}^{\circ }$.

**Assumption 2** *For constants c*_1_, *c*_2_ > 0,

- $\underset{\left\{A:|A|\leq 2\kappa_{\max}^{\circ }\right\}}{\min}\underset{\left\{\zeta :{\left\|\zeta_{A^{c}}\right\|}_{1}\leq 3{\left\|\zeta_{A}\right\|}_{1}\right\}}{\min}∥\text{X}\zeta {∥}_{2}^{2}/n∥\zeta {∥}_{2}^{2}\geq c_{1}.$
- $\underset{1\leq l\leq q}{\max}{\left(X^{⊤}X\right)}_{ll}/n\leq c_{2}^{2}.$

**Assumption 3** $\underset{{\text{V}}_{lj}\neq 0}{\min}\left|{\text{V}}_{lj}\right|\geq 100c_{1}^{-1}c_{2}\underset{1\leq j\leq p}{\max}\left({\text{Ω}}_{jj}^{-1/2}\right)\sqrt{\log(q)/n+\log(n)/n}$.

Assumption 2 is a common condition for proving the convergence rate of the Lasso (Bickel et al., 2009; Zhang et al., 2014). As a replacement of Assumption 1A, it is satisfied with a probability tending to one for isotropic subgaussian or bounded ***X*** (Rudelson and Zhou, 2013). Assumption 3, as an alternative to Assumption 1B, specifies the minimal signal strength over candidate interventions. Such a signal strength requirement is used for establishing the high-dimensional variable selection consistency (Fan et al., 2014; Loh and Wainwright, 2017; Zhao et al., 2018). Moreover, Assumption 3 can be further relaxed to a less intuitive condition, Assumption 5; see Appendix A.3 for details.

**Theorem 4 (Finite termination and consistency)** *Suppose Assumptions 1-3 are met with constants c*_1_ < 6*c*_2_, *and the machine precision* tol ≪ 1/*n is negligible. For* 1 ≤ *j* ≤ *p, if the tuning parameters* $\left(\kappa_{j},\tau_{j}\right)$ *of* Algorithm 1 *are suitably chosen such that*

$$\kappa_{j}=\kappa_{j}^{\circ },\;\frac{36c_{2}}{c_{1}}\sqrt{{\text{Ω}}_{jj}^{-1}\left(\frac{\log(q)}{n}+\frac{\log(n)}{n}\right)}\leq \tau_{j}\leq \frac{2}{5}\underset{V_{lj}\neq 0}{\min}\left|{\text{V}}_{lj}\right|,$$

*then for any γ_j_ such that* $\tau_{j}^{-1}{\left(32c_{2}^{2}{\text{Ω}}_{jj}^{-1}n^{-1}(\log(q)+\log(n))\right)}^{1/2}\leq \gamma_{j}\leq c_{1}/6$, *almost surely we have* Algorithm 1 *yields a global solution* ${\hat{V}}_{\cdot j}$ of (7) *in at most* $1+\left\lceil \log\left(\kappa_{\max}^{\circ }\right)/\log(4)\right\rceil$ *DC iterations when n is sufficiently large, where* ⌈·⌉ *is the ceiling function. Moreover, almost surely we have* Algorithm 2 *recovers* ${ℰ}_{+}$ *and* ${𝒥}_{+}$ *when n is sufficiently large*.

In view of Theorem 4 (proved in Appendix B.4), it suffices to specify the maximum number of DC iterations as $1+\left\lceil \log\left(\kappa_{\max}^{\circ }\right)/\log(4)\right\rceil$. Then the time complexity of Algorithm 1 is $p\times O\left(\log\kappa_{\max}^{\circ }\right)\times O\left(q^{3}+nq^{2}\right)$, where $O\left(q^{3}+nq^{2}\right)$ is that of solving a weighted Lasso (Efron et al., 2004). Note that Algorithm 2 does not involve heavy computation, so the overall time complexity for estimating the ARG (Algorithms 1–2) is $O\left(p\times \log\kappa_{\max}^{\circ }\times \left(q^{3}+nq^{2}\right)\right)$. Finally, the peeling method does not apply to observational data (**W** = **0**). In a sense, interventions are essential.

Theorem 4 establishes the consistent reconstruction by the peeling algorithm for the ARG. Yet, it does not provide any uncertainty measure for the presence of some directed edges in the true DAG. In what follows, we will develop an asymptotic theory for hypothesis tests concerning directed edges of interest.

### 4.2 Inferential theory

Given *H*_0_, let $𝒮=\left(Y,X;{ℰ}_{+}\cup 𝒟,{ℐ}_{+}\right)$.

**Assumption 4** $\underset{\left\{j:D_{𝒮}(j)\neq \emptyset \right\}}{\max}\left(\left|{\text{PA}}_{𝒮}(j)\right|+|{\text{IN}}_{𝒮}(j)|\right)/n\leq \rho \;\text{as}\;n\to \infty$, *for a constant* 0 < *ρ* < 1.

Assumption 4 is a hypothesis-specific condition restricting the underlying dimension of the testing problem. Usually, $\left|{\text{PA}}_{𝒮}(j)\right|≍\left|{\mathrm{AN}}_{𝒢}(j)\right|≍\left|{\text{IN}}_{𝒮}(j)\right|≍\kappa_{j}^{\circ }\ll p;1\leq j\leq p$, which relaxes the condition $n\gg p\log(p)\sqrt{\left|𝒟\right|}$ for the constrained likelihood ratio test (Li et al., 2020).

**Theorem 5 (Empirical p-values)** *Suppose Assumptions 1-4 are met and H*_0_ *is regular. Assume the tuning parameters in* Algorithm 1 *satisfy the requirements in Theorem 4*.

- *For the test of directed edges* (2),
$$\underset{\theta \;\text{satisfies}\;H_{0}\;\text{in}\;(2)}{\underset{n\to \infty }{\lim}}\underset{M\to \infty }{\lim}{\mathbb{P}}_{\theta }\left(\text{Pval}<\alpha \right)=\alpha ,\mathrm{if}H_{0}\;\mathrm{is nondegenerate}.\;\underset{\theta \;\text{satisfies}\;H_{0}\;\text{in}\;(2)}{\underset{n\to \infty }{\lim}}\underset{M\to \infty }{\lim}{\mathbb{P}}_{\theta }\left(\text{Pval}=1\right)=1,\mathrm{if}H_{0}\;\mathrm{is degenerate}.$$
- *For the test of directed pathways* (3),
$$\underset{\theta \;\text{satisfies}\;H_{0}\;\text{in}\;(3)}{\underset{n\to \infty }{\mathrm{lim sup}}}\underset{M\to \infty }{\lim}{\mathbb{P}}_{\theta }\left(\text{Pval}<\alpha \right)=\alpha ,\mathrm{if}H_{0}\;\mathrm{is nondegenerate with}|𝒟|=|ℋ|.\;\underset{\theta \;\text{satisfies}\;H_{0}\;\text{in}\;(3)}{\underset{n\to \infty }{\mathrm{lim sup}}}\underset{M\to \infty }{\lim}{\mathbb{P}}_{\theta }\left(\text{Pval=1}\right)=1,\mathrm{if}|𝒟|<|ℋ|.$$

By Theorem 5 (proved in Appendix B.5), the DP likelihood ratio test yields a valid p-value for (2) and (3) under appropriate conditions. Note that |𝒟| is permitted to depend on *n*. Moreover, Proposition 6 (proved in Appendix B.6) summarizes the asymptotics for directed edge test (2).

**Proposition 6 (Asymptotics of edge test)** *Suppose the assumptions of Theorem 5 are met. Under a nondegenerate and regular H*_0_, *as n* → ∞,

- $2\text{Lr}\overset{d}{\to }\chi_{\left|𝒟\right|}^{2},\;\text{if}\;|𝒟|>0$ *is fixed*.
- $\left(2\text{Lr}-|𝒟|)/\sqrt{2|𝒟|}\overset{d}{\to }N(0,1\right)$, *if* |𝒟|log(|𝒟|)/n→0.

**Remark 8** *As opposed to the entire* ${𝒢}_{+}$ *(or* 𝒮*), Theorem 5 and Proposition 6 only require correct identification of the local structures* ${\left\{{\text{AN}}_{{𝒢}_{+}}(j),{\text{IN}}_{{𝒢}_{+}}(j),{\text{D}}_{𝒮}(j)\right\}}_{\left\{j:{\text{D}}_{𝒮}(j)\neq \emptyset \right\}}$. *This requirement can be reasonably satisfied when the sample size is moderately large, as illustrated in* Section 5.

Next, we analyze the local limit power of the proposed tests for (2) and (3). Assume $\theta^{\circ }=\left(U^{\circ },W^{\circ }\right)$ satisfies *H*_0_. Let $\text{Δ}\in {\mathbb{R}}^{p\times p}$ satisfy ${\text{Δ}}_{{𝒟}^{c}}=0$ so that **U**° + **Δ** represents a DAG. For nondegenerate and regular *H*_0_, consider an alternative $H_{a}:{\text{U}}_{ℋ}={\text{U}}_{ℋ}^{\circ }+{\text{Δ}}_{ℋ}$, and define the power function as

(18) $$\beta \left(\theta^{\circ },\text{Δ}\right)={\mathbb{P}}_{H_{a}}(\text{Pval}<\alpha ).$$

**Proposition 7 (Local power of edge test)** *Suppose H*_0_ *is nondegenerate and regular. Let* ${∥\text{Δ}∥}_{F}={\left\|{\text{Δ}}_{ℋ}\right\|}_{F}=n^{-1/2}\delta$ *when* |𝒟|>0 *is fixed and* ${∥\text{Δ}∥}_{F}={\left|𝒟\right|}^{1/4}n^{-1/2}h$ *when* |𝒟|→∞, *where δ* > 0 *and* ‖⋅‖_*F*_ *is the matrix Frobenius norm. If the assumptions of Theorem 5 are met, then under H_a_, as n, M* → ∞,

$$\beta \left(\theta^{\circ },\text{Δ}\right)\geq \{\begin{array}{l} {\mathbb{P}}_{Z∼N\left(0,I_{\left|𝒟|\times |𝒟\right|}\right)}\left({\left\|Z+c_{l}\sqrt{n}\text{Δ}\right\|}_{2}^{2}>\chi_{|𝒟|,1-\alpha }^{2}\right)\;if\;|𝒟|>0\;is\;fixed,\\ {\mathbb{P}}_{Z∼N(0,1)}\left(Z>z_{1-\alpha }-c_{l}∥\text{Δ}{∥}_{2}^{2}/\sqrt{2|𝒟|}\right)\;\;if\;|𝒟|\to \infty ,\frac{\left|𝒟|\log|𝒟\right|}{n}\to 0, \end{array}$$

*where* $\chi_{|𝒟|,1-\alpha }^{2}$ *and z*_1−*α*_ *denote the* (1 − *α*)*th quantile of distributions* $\chi_{\left|𝒟\right|}^{2}$ *and N*(0, 1), *respectively. Hence,* $\underset{\delta \to \infty }{\lim}\;\underset{n\to \infty }{\lim}\beta \left(\theta^{\circ },\text{Δ}\right)=1$.

**Proposition 8 (Local power of pathway test)** *Suppose H*_0_ *is nondegenerate and regular with* |𝒟|=|ℋ|. *Let* $\min_{(k,j)\in ℋ}\left|{\text{U}}_{kj}^{\circ }+{\text{Δ}}_{kj}\right|=n^{-1/2}\delta$ *when* |ℋ|>0 *is fixed and* $\min_{(k,j)\in ℋ}\left|{\text{U}}_{kj}^{\circ }+{\text{Δ}}_{kj}\right|=n^{-1/2}\delta \sqrt{\log|ℋ|}$ *when* |ℋ|→∞. *If the assumptions of Theorem 5 are met, then under H_a_, as n, M* → ∞,

$$\beta \left(\theta^{\circ },\text{Δ}\right)\geq 1-\frac{\left|ℋ\right|}{\sqrt{2\pi }}\exp\left(-\frac{1}{2}{\left(\delta \sqrt{\log|ℋ|}/\underset{1\leq j\leq p}{\max}{\text{Ω}}_{jj}-\sqrt{\chi_{1,1-\alpha }^{2}}\right)}^{2}\right),$$

*where* $\chi_{1,1-\alpha }^{2}$ *is the* (1 − *α*)*th quantile of distribution* $\chi_{1}^{2}$. *Hence*, $\underset{\delta \to \infty }{\lim}\;\underset{n\to \infty }{\lim}\;\beta \left(\theta^{\circ },\text{Δ}\right)=1$.

The proofs of Propositions 7 and 8 are deferred to Appendix B.7 and B.8.

## 5. Simulations

This section investigates the operating characteristics of the proposed tests and the peeling algorithm via simulations. In simulations, we consider two setups for generating $U\in {\mathbb{R}}^{p\times p}$, representing random and hub DAGs, respectively.

- **Random graph.** The upper off-diagonal entries U*_kj_*; *k* < *j* are sampled independently from {0, 1} according to Bernoulli(1/*p*), while other entries are zero. This generates a random graph with a sparse neighborhood.
- **Hub graph.** Set U_1,2*j*+1_ = 1 and U_2,2*j*+2_ = 1 for *j* = 1, … , ⌊*p*/2⌋ − 2, while other entries are zero. This generates a hub graph, where nodes 1 and 2 are hub nodes with a dense neighborhood.

Moreover, we consider three setups for intervention matrix $W\in {\mathbb{R}}^{q\times p}$, representing different scenarios. Setups (A) and (B) are designed for inference, whereas Setup (C) in Section 5.2 is designed to compare with the method of Chen et al. (2018) for structure learning. Let $W={\left(A^{⊤},B^{⊤},0^{⊤}\right)}^{⊤}$, where $A,B\in {\mathbb{R}}^{p\times p}$ and $0\in {\mathbb{R}}^{(q-2p)\times p}$.

- **Setup (A).** Set ${\text{A}}_{jj}={\text{B}}_{jj}={\text{B}}_{j,j+1}=1;j=1,\ldots ,p-1,{\text{A}}_{pp}=1$, while other entries of **A, B** are zero. Then, *X*_1_, … , *X_p_* are instruments for *Y*_1_, … , *Y_p_*, respectively, *X*_*p*+1_, … , *X*_2*p*−1_ are invalid instruments with two targets, and *X*_2*p*_, … , *X_q_* represent inactive interventions.
- **Setup (B).** Set ${\text{A}}_{jj}={\text{A}}_{j,j+1}={\text{B}}_{jj}={\text{B}}_{j,j+1}=1;j=1,\ldots ,p-1,{\text{A}}_{pp}=1$, while other entries of **A, B** are zero. Here, the only valid instrument is *X_p_* on *Y_p_*, and the other intervention variables either have two targets or are inactive. Importantly, Assumption 1C is not met.

To generate (***Y , X***) for each setup, we sample $X∼N\left(0,{\text{Σ}}_{X}\right)$ with ${\left({\text{Σ}}_{X}\right)}_{{ll}^{\prime }}=0.5^{\left|l-l^{\prime }\right|};1\leq l,l^{\prime }\leq q$ and sample ***Y*** according to (1) with $\left(U,W,\sigma_{1}^{2},\ldots ,\sigma_{p}^{2}\right)$, where $\sigma_{1}^{2},\ldots ,\sigma_{p}^{2}$ are set to be equally spaced from 0.5 to 1.

### 5.1 Inference

We compare three tests in empirical type-I errors and powers in simulated examples, namely, the DP likelihood ratio test (DP-LR) in Algorithm 3, the asymptotic likelihood ratio test (LR), and the oracle likelihood ratio test (OLR). Here LR uses Lr, while OLR uses $\mathrm{Lr}(𝒮,\hat{\text{Σ}})$ assuming that 𝒮 were known in advance. The p-values of LR and OLR are computed via Proposition 6. The implementation details of these tests are in Appendix C.1.

For the empirical type-I error of a test, we compute the percentage of times rejecting *H*_0_ out of 500 simulations when *H*_0_ is true. For the empirical power of a test, we report the percentage of times rejecting *H*_0_ out of 100 simulations when *H_a_* is true under alternative hypotheses *H_a_*.

- **Test of directed edges.** For (2), we examine two different hypotheses:
  - $H_{0}:{\text{U}}_{1,20}=0\;\text{versus}\;H_{a}:{\text{U}}_{1,20}\neq 0$. In this case, |𝒟|=1.
  - $H_{0}:U_{ℋ}=0\;\text{versus}\;H_{a}:U_{ℋ}\neq 0$, where ℋ={(k,20):k=1,…,15}. In this case, |𝒟|=15.
  Moreover, five alternatives $H_{a}:{\text{U}}_{1,20}=0.1l$ and ${\text{U}}_{ℋ\setminus \{(1,20)\}}=0;l=1,2,3,4,5$, are used for the power analysis in (i) and (ii). The data are generated by modifying **U** accordingly.
- **Test of directed pathways.** We test the directed path $Y_{1}\to Y_{5}\to Y_{10}\to Y_{15}\to Y_{20}$, namely ℋ={(1,5),(5,10),(10,15),(15,20)} in (3). Since (3) is a test of composite null hypothesis, the data are generated under a graph with parameters (**U, W, Σ**) satisfying *H*_0_, where ${\text{U}}_{ℋ}=0$. Five hypotheses $H_{a}:{\text{U}}_{ℋ}=0.1l;l=1,2,3,4,5$, are used for the power analysis.

**Figure 2:**
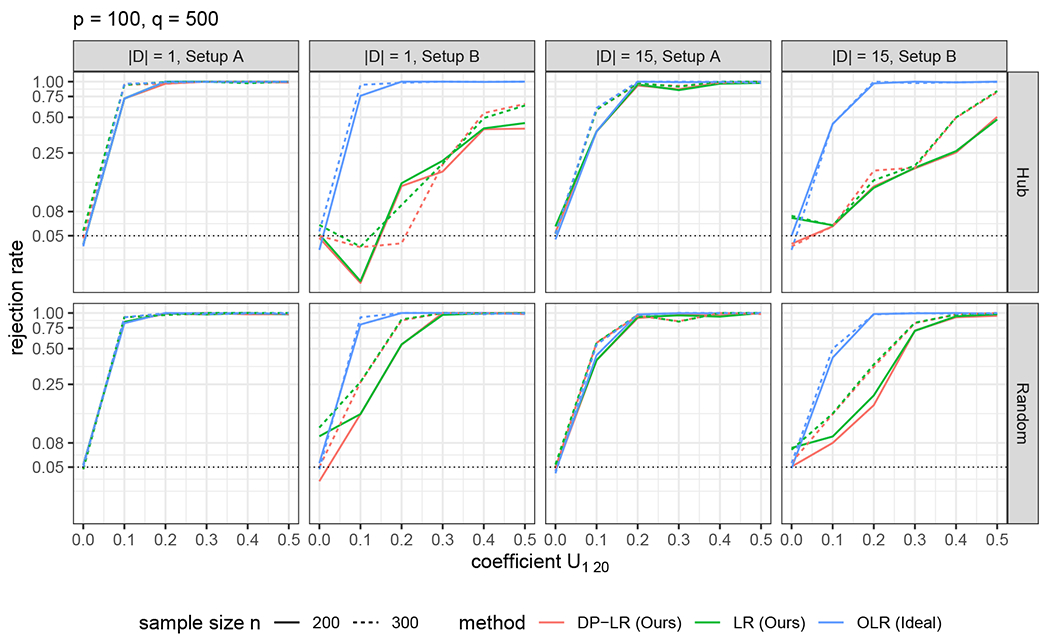
Empirical type-I errors and powers of tests of directed edges. The black dotted line marks the nominal level of significance *α* = 0.05.

For testing directed edges, as displayed in Figure 2, DP-LR and LR perform well compared to the ideal test OLR in Setup (A) with Assumption 1 satisfied. In Setup (B) with Assumption 1C not fulfilled, DP-LR appears to have control of type-I error, whereas LR has an inflated empirical type-I error compared to the nominal level *α* = 0.05. This discrepancy is attributed to the data perturbation scheme accounting for the uncertainty of identifying *S*. However, both suffer a loss of power compared to the oracle test OLR in this setup. This observation suggests that without Assumption 1C, the peeling algorithm tends to yield an estimate ${\hat{𝒢}}_{+}⊇{𝒢}_{+}$, which overestimates ${𝒢}_{+}$, resulting in a power loss.

**Figure 3:**
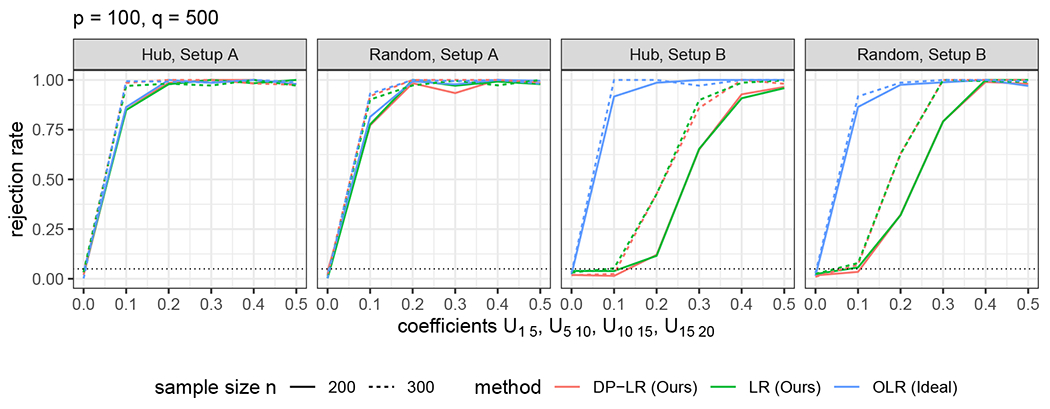
Empirical type-I errors and powers of tests of a directed pathway. The black dotted line marks the nominal level of significance *α* = 0.05.

For testing directed pathways, as indicated in Figure 3, we observe similar phenomena as in the previous directed edge tests. Of note, both LR and DP-LR are capable in controlling type-I error of directed path tests.

In summary, DP-LR has a suitable control of type-I error when there are invalid instruments and Assumption 1C is violated. Concerning the power, DP-LR and LR are comparable in all scenarios and their powers tend to one as the sample size *n* or the signal strength of tested edges increases. Moreover, DP-LR and LR perform nearly as well as the oracle test OLR when Assumption 1 is satisfied. These empirical findings agree with our theoretical results.

### 5.2 Structure learning

This subsection compares the peeling algorithm with the Two-Stage Penalized Least Squares (2SPLS, Chen et al. (2018)) in terms of the structure learning accuracy. For peeling, we consider Algorithm 2 with an additional step (9) for structure learning of **U**. For 2SPLS, we use the R package BigSEM.

2SPLS requires that all the intervention variables to be target-known instruments in addition to Assumption 1C. Thus, we consider an additional Setup (C).

- **Setup (C).** Let $W={\left(I_{p\times p},0\right)}^{⊤}\in {\mathbb{R}}^{q\times p}$. Then *X*_1_, … , *X_p_* are valid instruments for *Y*_1_, … , *Y_p_*, respectively, and other intervention variables are inactive.

For 2SPLS, we assign each active intervention variable to its most correlated primary variable in Setups A-C. In Setup C, this assignment yields a correct identification of valid instruments, meeting all the requirements of 2SPLS.

**Figure 4:**
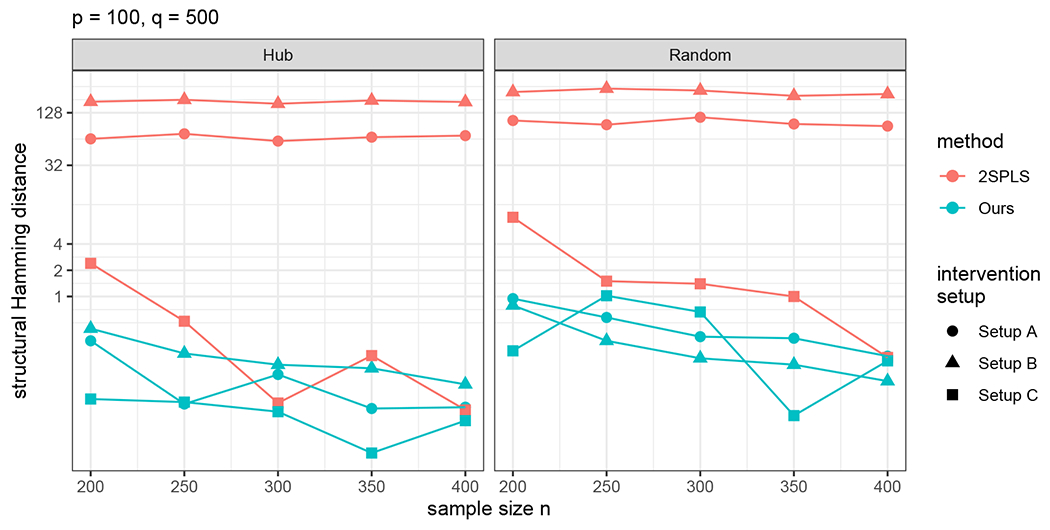
SHDs for the reconstructed DAG by the peeling algorithm and 2SPLS, where a smaller value of SHD indicates a better result.

For each scenario, we compute the structural Hamming distance (SHD)

$$\text{SHD}(\hat{\text{U}},\text{U})=\underset{k,j}{\sum }\left|\text{I}\left({\hat{\text{U}}}_{kj}\neq 0\right)-\text{I}\left({\text{U}}_{kj}\neq 0\right)\right|,$$

averaged over 100 runs. As shown in Figure 4, the peeling algorithm outperforms 2SPLS, especially when there are invalid instruments and Assumption 1C is violated.

Appendix C.2 contains additional numerical experiments on structure learning, including the results of different sparsity settings, SHD transition curves, and different numbers of interventions.

### 5.3 Comparison of inference and structure learning

This subsection compares the proposed DP testing method against the proposed structure learning method in (9) in terms of inferring the true graph structure. To this end, we consider Setup (A) in Section 5.1 with *p* = 30, *q* = 100, and the hypotheses

$$H_{0}:{\text{U}}_{1,20}=0\;\text{versus}\;H_{a}:{\text{U}}_{1,20}=1/\sqrt{n}.$$

For DP inference, we use *α* = 0.05 and choose the tuning parameters by BIC as in previous experiments; see Appendix C.1 for details. For structure learning, we reject the null hypothesis when ${\hat{\text{U}}}_{1,20}\neq 0$.

**Figure 5:**
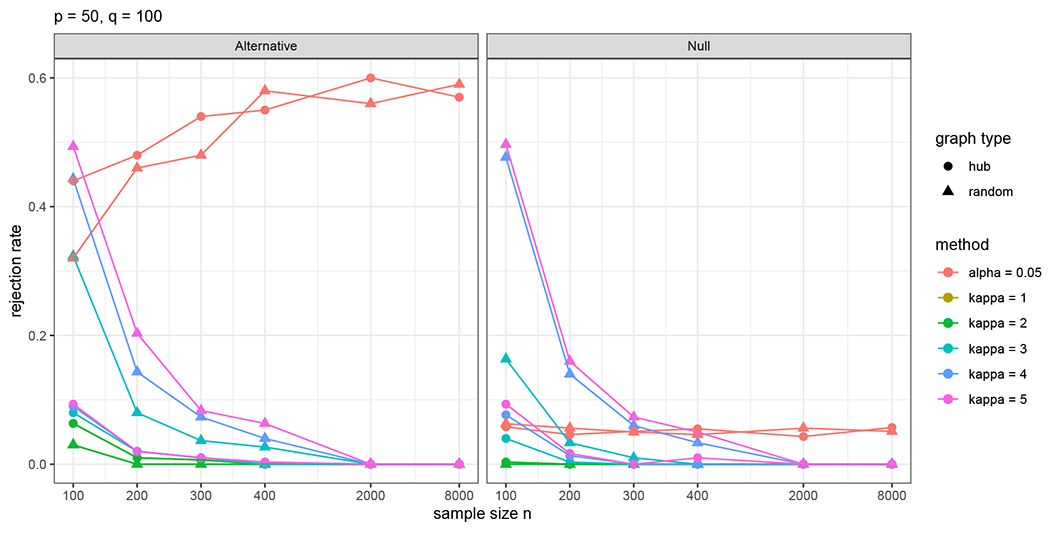
Rejection rates for *H*_0_ : U_1,20_ = 0 versus $H_{a}:{\text{U}}_{1,20}=1/\sqrt{n}$ by DP inference and structure learning. For structure learning, *H*_0_ is rejected if ${\hat{\text{U}}}_{1,20}\neq 0$. The red lines indicate the results of DP inference using the significance level *α* = 0.05. The other colored lines display the results of structure learning using different sparsity parameter values *κ* = 1, 2, 3, 4, 5. The simulation is repeated for 500 times and *κ* = 2 is chosen by BIC in over 90% cases.

As displayed in Figure 5, when the null hypothesis *H*_0_ is true, the DP testing method controls type-I error very close to the nominal level of 0.05, whereas the type-I error of the structure learning varies greatly depending on the tuning parameter selection. Under the alternative hypothesis *H*_a_**, the DP inference enjoys high statistical power than structure learning methods when *n* ≥ 200. Interestingly, the power of structure learning diminishes as *n* increases. This observation is in agreement with our theoretical results in Theorem 10, suggesting that consistent reconstruction requires the smallest size of nonzero coefficients to be of order $≳\sqrt{\log(n)/n}$ with the tuning parameter *τ* of the same order (fixing *p, q*). In this case, the edge U_1_,_20_ is of order $1/\sqrt{n}$, which is less likely to be reconstructed as *n* increases. In contrast, Proposition 7 indicates that a DP test has a non-vanishing power when the hypothesized edges are of order $1/\sqrt{n}$.

Figure 5 demonstrates some important distinctions between inference and structure learning. When different tuning parameters are used, the structure learning results correspond to different points on an ROC curve. Although it is asymptotically consistent when optimal tuning parameters are used, structure learning lacks an uncertainty measure of graph structure identification. As a result, it is nontrivial for structure learning methods to trade-off the false discovery rate and detection power in practice. This makes the interpretation of such results hard, especially when they heavily rely on hyperparameters as in Figure 5. By comparsion, DP inference aims to maximize statistical power while controlling type-I error at a given level, offering a clear interpretation of its result. This observation agrees with the discussions in the literature on variable selection and inference (Wasserman and Roeder, 2009; Meinshausen and Bühlmann, 2010; Lockhart et al., 2014; Candes et al., 2018) and it justifies the demand for inferential tools for directed graphical models.

## 6. ADNI data analysis

This section applies the proposed tests to analyze an Alzheimer’s Disease Neuroimaging Initiative (ADNI) dataset. In particular, we infer gene pathways related to Alzheimer’s Disease (AD) to highlight some gene-gene interactions differentiating patients with AD/cognitive impairments and healthy individuals.

The raw data are available in the ADNI database (https://adni.loni.usc.edu), including gene expression, whole-genome sequencing, and phenotypic data. After cleaning and merging, we have a sample size of 712 subjects. From the KEGG database (Kanehisa and Goto, 2000), we extract the AD reference pathway (hsa05010, https://www.genome.jp/pathway/hsa05010), including 146 genes in the ADNI data.

For data analysis, we first regress the gene expression levels on five covariates – gender, handedness, education level, age, and intracranial volume, and then use the residuals as gene expressions in the following analysis. Next, we extract the genes with at least one SNP at a marginal significance level below 10^−3^, yielding *p* = 63 genes as primary variables. For these genes, we further extract their marginally most correlated two SNPs, resulting in *q* = 63 × 2 = 126 SNPs as unspecified intervention variables, in subsequent data analysis. All gene expression levels are normalized.

The dataset contains individuals in four groups, namely, Alzheimer’s Disease (AD), Early Mild Cognitive Impairment (EMCI), Late Mild Cognitive Impairment (LMCI), and Cognitive Normal (CN). For our purpose, we treat 247 CN individuals as controls while the remaining 465 individuals as cases (AD-MCI). Then, we use the gene expressions and the SNPs to reconstruct the ancestral relations and infer gene pathways for 465 AD-MCI and 247 CN control cases, respectively.

**Figure 6:**
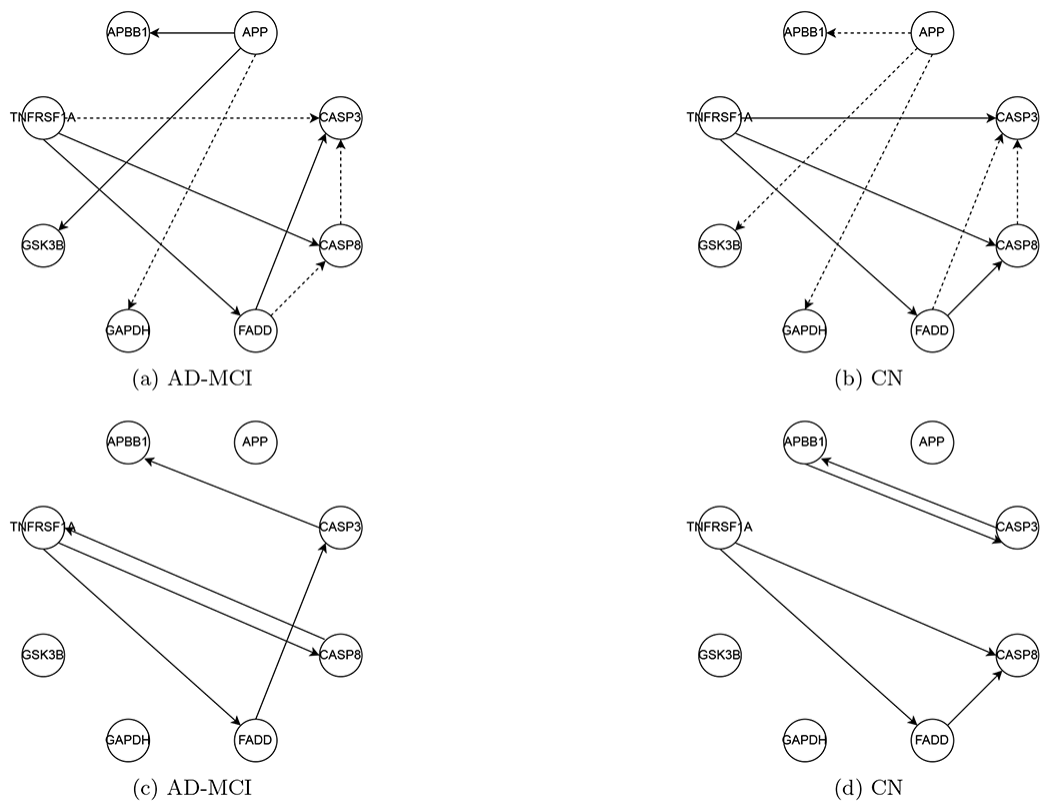
Display of the subnetworks associated with genes APP and CASP3. (a) and (b): Solid/dashed arrows indicate significant/insignificant edges at *α* = 0.05 after adjustment for multiplicity by the Bonferroni-Holm correction. (c) and (d): Solid arrows indicate the reconstructed edges using 2SPLS (Chen et al., 2018).

**Figure 7:**
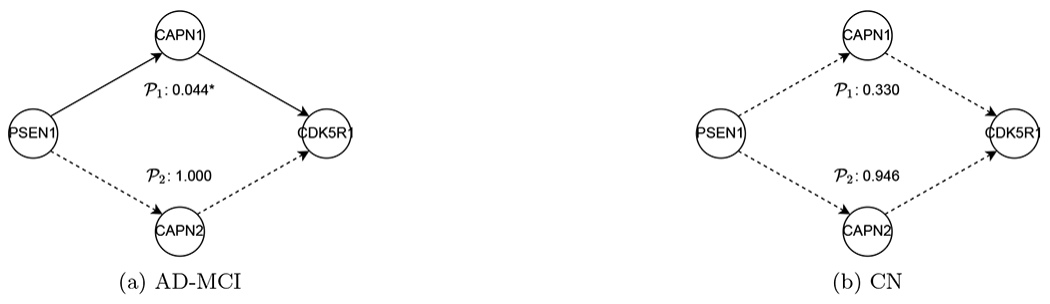
The p-values of pathway tests (3) by the proposed tests for the AD-MCI and CN groups, where p-values are adjusted for multiplicity by the Bonferroni-Holm correction and solid/dashed arrows indicate significant/insignificant pathways at *α* = 0.05.

In the literature, genes APP, CASP3, and PSEN1 are well-known to be associated with AD, reported to play different roles in AD patients and healthy subjects (Julia and Goate, 2017; Su et al., 2001; Kelleher III and Shen, 2017). For this dataset, we conduct hypothesis testing on edges and pathways related to genes APP, CASP3, and PSEN1 in the KEGG AD reference (hsa05010) to evaluate the proposed DP inference by checking if DP inference can discover the differences that are reported in the biomedical literature. First, we consider testing *H*_0_ : U_*kj*_ = 0 versus $H_{a}:{\text{U}}_{kj}\neq 0$, for each edge (*k, j*) as shown in Figure 6 (a) and (b). Moreover, we consider two hypothesis tests of pathways $H_{a}:{\text{U}}_{kj}=0$ for some $(k,j)\in {𝒫}_{\ell }$ versus *H_a_* : *U_kj_* ≠ 0 for all $(k,j)\in {𝒫}_{\ell }$; ℓ = 1, 2, where the two pathways are specified by ${𝒫}_{1}=\{\text{PSEN1}\to \text{CAPN1}\to \text{CDK5R1}\}$, and ${𝒫}_{2}=\{\text{PSEN1}\to \text{CAPN2}\to \text{CDK5R1}\}$. See Figure 7. Of note, for clear visualization, Figure 6 (a), (b) and Figure 7 only display the edges related to hypothesis testing, whereas the ancestral relations are reconstructed using *p* = 63 genes and *q* = 126 SNPs for AD-MCI and CN groups separately.

**Figure 8:**
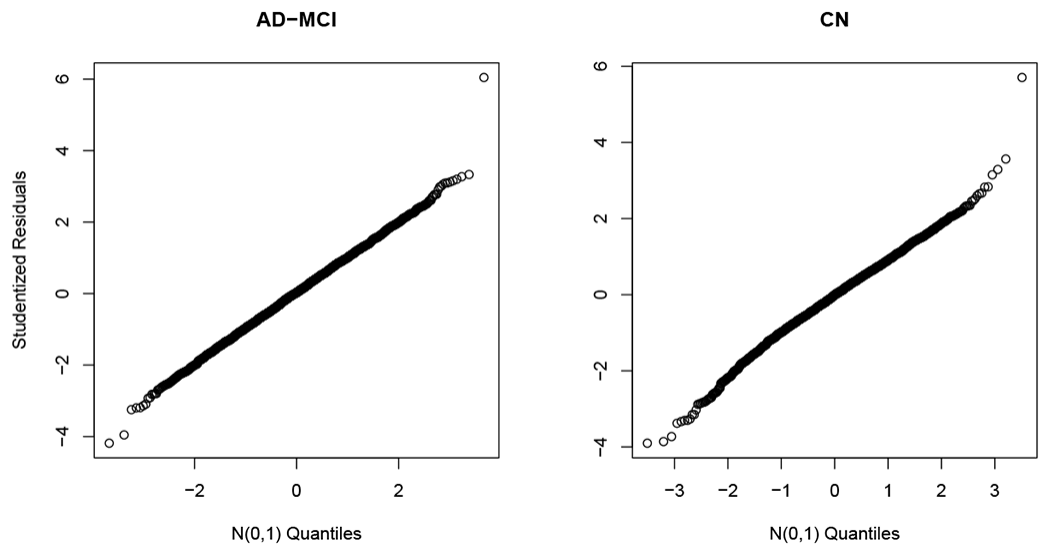
Normal quantile-quantile plots of studentized residuals of the AD-MCI and CN groups.

In Figures 6–7, the significant results under the level *α* = 0.05 after the Holm-Bonferroni adjustment for 2 × (9 + 2) = 22 tests are displayed. In Figures 6, the edge test in (2) exhibits a strong evidence for the presence of directed connectivity {APP → APBB1, APP → GSK3B, FADD → CASP3} in the AD-MCI group, but no evidence in the CN group. Meanwhile, this test suggests the presence of connections {TNFRSF1A → CASP3, FADD → CASP8} in the CN group but not so in the AD-MCI group. In both groups, we identify directed connections {TNFRSF1A → FADD, TNFRSF1A → CASP8}. In Figure 7, the pathway test (3) supports the presence of a pathway PSEN1 → CAPN1 → CDK5R1 in the AD-MCI group with a p-value of 0.044 but not in the CN group with a p-value of 0.33. The pathway PSEN1 → CAPN2 → CDK5R1 appears insignificant at *α* = 0.05 for both groups. Also noted is that some of our discoveries agree with the literature according to the AlzGene database (alzgene.org) and the AlzNet database (https://mips.helmholtz-muenchen.de/AlzNet-DB). Specifically, GSK3B differentiates AD patients from normal subjects; as shown in Figures 6, our result indicates the presence of connection APP → GSK3B for the AD-MCI group, but not for the CN group, the former of which is confirmed by Figure 1 of Kremer et al. (2011). The connection APP → APBB1 also differs in AD-MCI and CN groups, which appears consistent with Figure 3 of Bu (2009). Moreover, the connection CAPN1 → CDK5R1, in the pathway PSEN1 → CAPN1 → CDK5R1 discovered in AD-MCI group, is found in the AlzNet database (interaction-ID 24614, https://mips.helmholtz-muenchen.de/AlzNet-DB/entry/show/1870). Finally, as suggested by Figure 8, the normality assumption in (1) is adequate for both groups.

By comparison, as shown in Figure 6 (c) and (d), gene APP in the reconstructed networks by 2SPLS (Chen et al., 2018) is not connected with other genes, indicating no regulatory relation of APP with other genes in the AD-MCI and CN groups. However, as a well-known gene associated with AD, APP is reported to play different roles in controlling the expressions of other genes for AD patients and healthy people (Matsui et al., 2007; Julia and Goate, 2017). Our results in Figure 6 (a) and (b) are congruous with the studies: the connections of APP with other genes are different in our estimated networks for AD-MCI and CN groups.

In summary, our findings seem to agree with those in the literature (Julia and Goate, 2017; Su et al., 2001; Kelleher III and Shen, 2017), where the subnetworks of genes APP, CASP3 in Figure 6 and PSEN1 in Figure 7 differentiate the AD-MCI from the CN groups. Furthermore, the pathway PSEN1 → CAPN1 → CDK5R1 in Figure 7 seems to differentiate these groups, which, however, requires validation in biological experiments.

## 7. Summary

This article proposes structure learning and inference methods for a Gaussian DAG with interventions, where the targets and strengths of interventions are unknown. A likelihood ratio test is derived based on an estimated ARG formed by ancestral relations and candidate interventional relations. This test accounts for the statistical uncertainty of the construction of the ARG based on a novel data perturbation scheme. Moreover, we develop a peeling algorithm for the ARG construction. The peeling algorithm allows scalable computing and yields a consistent estimator. The numerical studies justify our theory and demonstrate the utility of our methods.

The proposed methods can be extended to many practical situations beyond biological applications with independent and identically distributed data. An instance is to infer directed relations between multiple autoregressive time series (Pamfil et al., 2020), where the lagged variables and covariates can serve as interventions for each time series.

The current work has two limitations. First, the inferential theory requires (asymptotically) correct recovery of the local DAG structures (Remark 8) to produce valid p-values, similar to Shi et al. (2019) and Zhu et al. (2020). As illustrated in numerical studies, the graph structures are reasonably recovered when *n* is moderately large, and the DP scheme empirically alleviates the issue of inference after the ARG reconstruction. However, whether valid p-values can be obtained without the exact reconstruction of nuisance graph structures remains unclear in theory. Second, the proposed methods do not treat hidden confounding, which often arises in practice and can bias the results of both inference and learning. One future research direction is to extend the framework of unspecified interventions to allow unmeasured confounders.

## Figures and Tables

**Figure 9:**
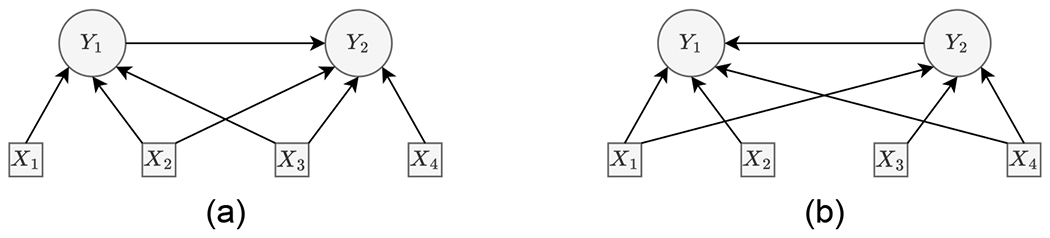
(a) Display of DAG defined by (19). (b) Display of DAG defined by (20).

**Figure 10:**
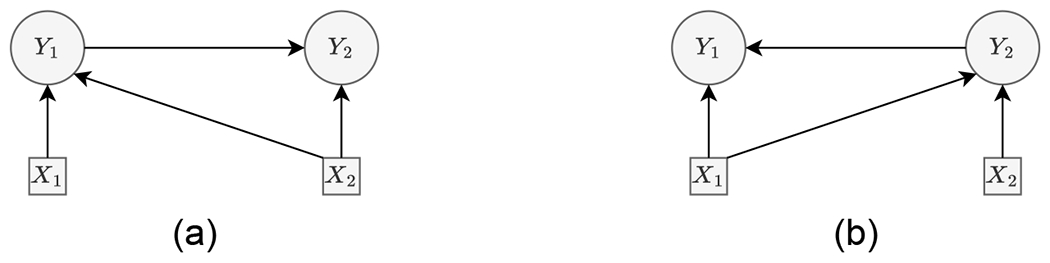
(a) Display of DAG defined by (21). (b) Display of DAG defined by (22).

**Figure 11:**
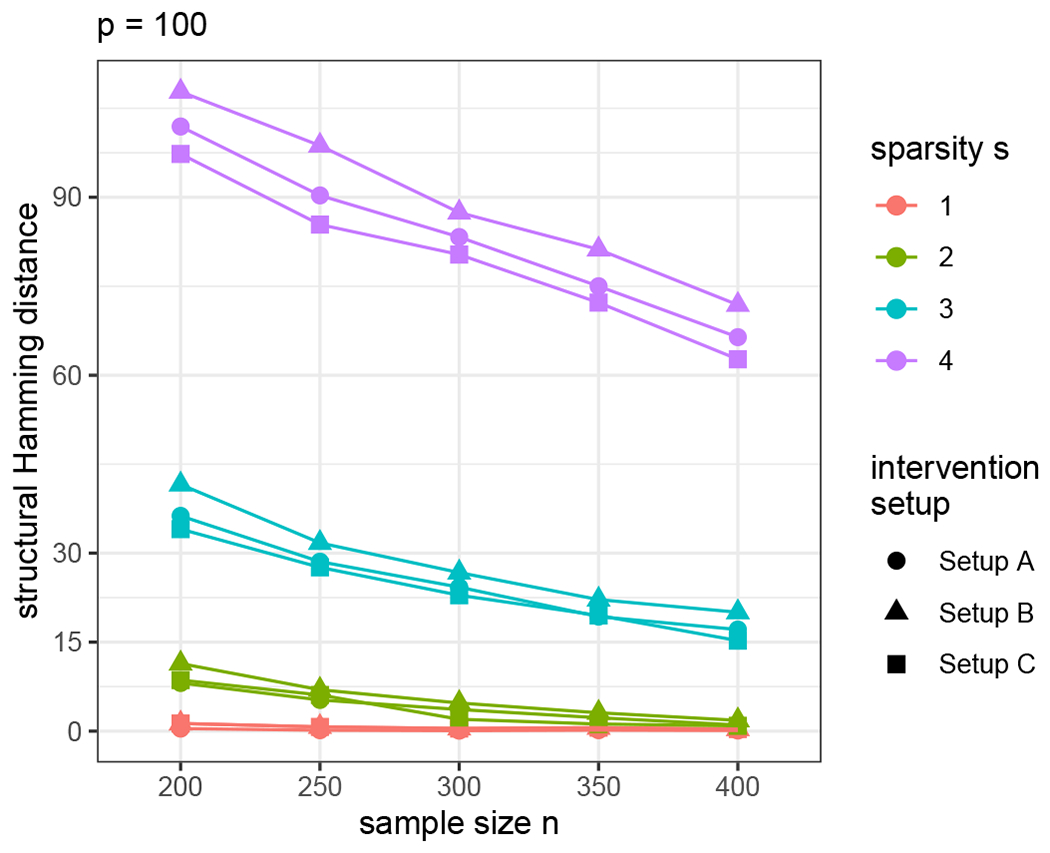
SHDs for the reconstructed DAG by the peeling algorithm.

**Figure 12:**
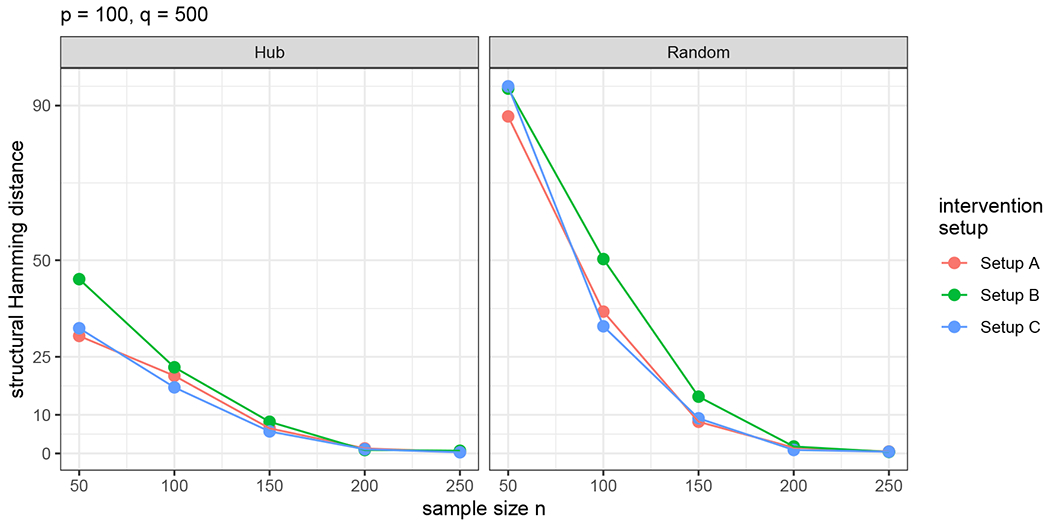
SHDs for the reconstructed DAG by the peeling algorithm. The experiment settings are the same as the ones in Section 5.2.

**Figure 13:**
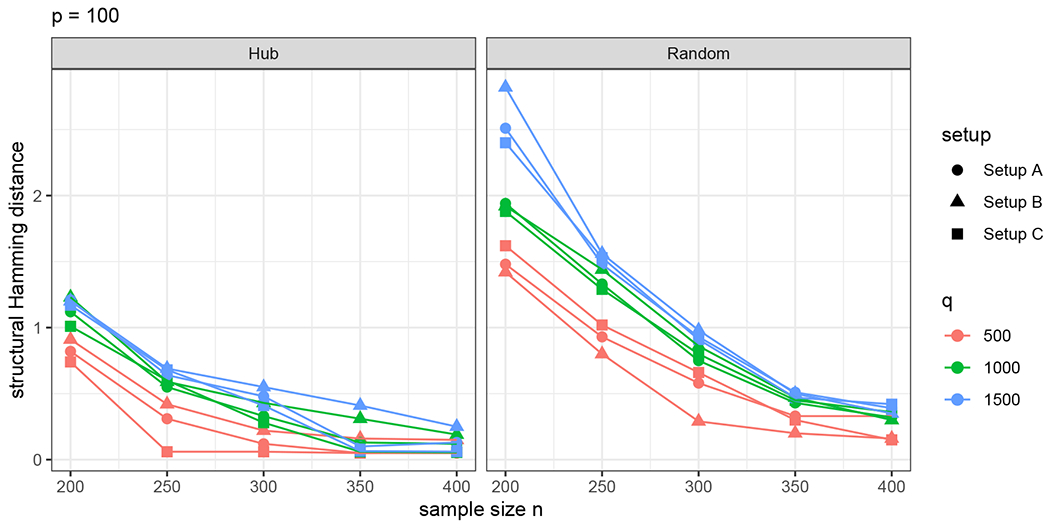
SHDs for the reconstructed DAG by the peeling algorithm. The experiment settings are the same as the ones in Section 5.2.

## Appendix A. Illustrative examples and discussions

### A.1 Identifiability of model (1) and Assumption 1

The parameter space for model (1) is

$$\left\{(U,W,\Sigma ):U\in {\mathbb{R}}^{p\times p}\;\text{represents a DAG},W\in {\mathbb{R}}^{p\times q},\Sigma =\text{diag}\left(\sigma_{1}^{2},\ldots ,\sigma_{p}^{2}\right)\right\}.$$

As suggested by Proposition 1, Assumption 1 (1A-1C) suffices for identification of every parameter value in the parameter space. Next, we show by examples that if Assumption 1B or 1C is violated then model (1) is no longer identifiable. To proceed, we rewrite (1) as

$$Y=V^{⊤}X+\varepsilon_{V},\;\varepsilon_{V}={\left(I-U^{⊤}\right)}^{-1}\varepsilon ∼N\left(0,{\text{Ω}}^{-1}\right),$$

where $\Omega =(I-U)\Sigma^{-1}\left(I-U^{⊤}\right)$ is a precision matrix and **V** = **W**(**I** − **U**)^−1^.

**Example 2 (Identifiability)** *In* model (1), *consider two non-identifiable bivariate situations: (1) p* = 2 *and q* = 4 *and (2) p* = 2 *and q* = 2.

1. Model (1) *is non-identifiable when Assumption 1B breaks down. Consider two different models with different parameter values:*
  (19)
  $$\theta :\;\;Y_{1}=X_{1}+X_{2}+X_{3}+\varepsilon_{1},\;\;Y_{2}=Y_{1}-X_{2}+X_{3}+X_{4}+\varepsilon_{2},$$
  (20)
  $$\tilde{\theta }:\;Y_{1}=0.5Y_{2}+0.5X_{1}+X_{2}-0.5X_{4}+{\tilde{\varepsilon }}_{1},\;Y_{2}=X_{1}+2X_{3}+X_{4}+{\tilde{\varepsilon }}_{2},$$
  *where* $\varepsilon_{1},\varepsilon_{2}∼N(0,1)$ *are independent, and* ${\tilde{\varepsilon }}_{1}∼N(0,0.5),{\tilde{\varepsilon }}_{2}∼N(0,2)$ *are independent. As depicted in* Figure 9, (19) *satisfies Assumption 1C. However, Assumption 1B is violated given that* $\text{Cov}\left(Y_{2},X_{2}|X_{\left\{1,3,4\right\}}\right)=0$ *and X*_2_ *is an intervention variable of Y*_2_. *This is because the direct interventional effect of X*_2_ *on Y*_2_ *are canceled out by its indirect interventional effect through Y*_1_. *Similarly*, (20) *satisfies Assumption 1C but* $\text{Cov}\left(Y_{1},X_{4}|X_{\left\{1,2,3\right\}}\right)=0$ *violating Assumption 1B. In this case, it can be verified that **θ** and* $\tilde{\theta }$ *correspond to the same distribution* ℙ(Y∣X), *because they share the same* (**V, Ω**) *even with different values of* (**U, W, Σ**). *Hence, it is impossible to infer the directed relation between Y*_1_ *and Y*_2_.
2. Model (1) *is non-identifiable when Assumption 1C breaks down. Consider two different models with different parameter values:*
  (21)
  $$\theta :\;\;Y_{1}=X_{1}+X_{2}+\varepsilon_{1},\;Y_{2}=Y_{1}+X_{2}+\varepsilon_{2},$$
  (22)
  $$\tilde{\theta }:\;Y_{1}=0.5Y_{2}+0.5X_{1}+{\tilde{\varepsilon }}_{1},\;Y_{2}=X_{1}+2X_{2}+{\tilde{\varepsilon }}_{2},$$
  *where* $\varepsilon_{1},\varepsilon_{2}∼N(0,1)$ *are independent, and* ${\tilde{\varepsilon }}_{1}∼N(0,0.5),{\tilde{\varepsilon }}_{2}∼N(0,2)$ *are independent. Note that* (21) *and* (22) *satisfy Assumption 1B. In* (21), *Y*_2_ *does not have any instrumental intervention although it has an invalid instrument X*_2_. *Similarly, in* (22), *neither does Y*_1_ *have any instrumental intervention while having an invalid instrument X*_1_. *As in the previous case, **θ** and* $\tilde{\theta }$ *yield the same distribution* ℙ(Y∣X) *because they share the same* (**V, Ω**) *even with different values of* (**U, W, Σ**). *In this case, it is impossible to infer the directed relation between Y*_1_ *and Y*_2_.

### A.2 Illustration of Algorithm 2

We now illustrate Algorithm 2 by Example 3.

**Example 3** *Consider* model (1) *with p* = *q* = 5,

(23)
$$\begin{array}{lll} Y_{1}=X_{1}+\varepsilon_{1}, & Y_{2}=0.5Y_{1}+X_{3}+\varepsilon_{2}, & Y_{5}=X_{4}+\varepsilon_{5},\\ Y_{3}=0.5Y_{2}+X_{5}+\varepsilon_{3}, & Y_{4}=0.5Y_{3}-0.1Y_{1}+X_{2}+\varepsilon_{4}, \end{array}$$

*where* $\varepsilon_{1},\ldots ,\varepsilon_{5}∼N(0,1)$ *independently. Then* (23) *defines a DAG as displayed in* Figure 1. *For illustration, we generate a random sample of size n* = 40 *and compute* $\hat{V}$ *by* Algorithm 1. *In particular*,

$$V=\left(\begin{array}{ccccc} 1 & 0.5 & 0.25 & 0.025 & 0\\ 0 & 0 & 0 & 1 & 0\\ 0 & 1 & 0.5 & 0.25 & 0\\ 0 & 0 & 0 & 0 & 1\\ 0 & 0 & 1 & 0.5 & 0 \end{array}\right),\;\hat{V}=\left(\begin{array}{ccccc} 0.92 & 0.48 & 0.27 & 0 & 0\\ 0 & 0 & 0 & 1.08 & 0\\ 0 & 1.03 & 0.52 & 0.21 & 0\\ 0 & 0 & 0 & 0 & 1.06\\ 0 & 0 & 0.98 & 0.55 & 0 \end{array}\right).$$

Algorithm 2 *proceeds as follows*.

- ${𝒱}_{Y}=\{1,2,3,4,5\}$ *: The interventions indexed by* ℬ={2,4} *are instruments on the leaves that are indexed by* 𝒧={4,5}.
  - *X*_2_ *is identified as an instrument of leaf node Y_4_ (X_2_ → Y_4_) because* ${\hat{\text{V}}}_{24}\neq 0$ *is the only nonzero in the row 2 with the smallest (positive) row ℓ*_0_-*norm*.
  - *X*_4_ *is identified as an instrument of leaf node Y*_5_ *(X*_4_ → *Y*_5_*) because* ${\hat{\text{V}}}_{45}\neq 0$ *is the only nonzero in row 4 with the smallest (positive) row ℓ*_0_-*norm*.
  *Then* {*Y*_4_, *Y*_5_} *are removed.*
- ${𝒱}_{Y}=\{1,2,3\}$*: The intervention indexed by* ℬ={5} *is an instrument on the leaf indexed by* 𝒧={3}.
  - *X*_5_ *is identified as an instrument of a leaf node Y*_3_ *(X*_5_ → *Y*_3_*) in* ${𝒢}^{\mathrm{work}}$ *given that* ${\hat{\text{V}}}_{53}\neq 0$ *is the only nonzero element in the row with the smallest (positive) row ℓ*_0_-*norm of the submatrix for Y*_1_, *Y*_2_, *Y*_3_.
  *Since Y*_3_ *is a leaf in* ${𝒢}^{\mathrm{work}}$, *Y*_4_ *has been removed, X*_5_ *is the only instrument on Y*_3_, *and* ${\hat{\text{V}}}_{54}\neq 0$, *we have* $(3,4)\in {\hat{ℰ}}_{+}$ *by Proposition 3. Then* {*Y*_3_} *is removed.*
- ${𝒱}_{Y}=\{1,2\}$*: The intervention indexed by* ℬ={3} *is an instrument on the leaf indexed by* 𝒧={2}.
  - *X*_3_ *is identified as an instrument of a leaf node Y*_2_ *(X*_3_ → *Y*_2_*) similarly in* ${𝒢}^{\mathrm{work}}$ *given that* ${\hat{\text{V}}}_{32}\neq 0$ *is the largest nonzero element in its row of the submatrix.*
  *Since Y*_2_ *is a leaf in* ${𝒢}^{\mathrm{work}}$, *Y*_3_ *has been removed*, *X*_3_ *is the only instrument on Y*_2_, *and* ${\hat{\text{V}}}_{33}\neq 0$, *we have* $(2,3)\in {\hat{ℰ}}_{+}$. *Then* {*Y*_2_} *is removed.*
- ${𝒱}_{Y}=\{1\}$*: The intervention indexed by* ℬ={1} *is an instrument on the leaf indexed by* 𝒧={1}.
  - *X*_1_ *is an instrument of Y*_1_ (*X*_1_ → *Y*_1_).
  *Since Y*_1_ *is a leaf node in* ${𝒢}^{\mathrm{work}}$, *Y*_2_ *has been removed, X*_1_ *is the only instrument on Y*_1_, *and* ${\hat{\text{V}}}_{12}\neq 0$, *we have* $(1,2)\in {\hat{ℰ}}_{+}$. *Then* {*Y*_1_} *is removed, and the peeling process is terminated*.

*Finally, Steps 9 and 10 identify*

$${\hat{ℰ}}_{+}=\{(1,2),(2,3),(3,4),(1,3),(1,4),(2,4)\},$$

$${\hat{ℐ}}_{+}=\{(1,1),(1,2),(1,3),(1,4),(2,4),(3,2),(3,3),(3,4),(4,5),(5,3),(5,4)\},$$

*which are equal to* ${ℰ}_{+}$ *and* ${ℐ}_{+}$, *respectively*.

In Example 3 $\left\{(l,j):{\hat{\text{V}}}_{lj}\neq 0\right\}\neq \left\{(l,j):{\text{V}}_{lj}\neq 0\right\}$, suggesting that the selection consistency of $\hat{\text{V}}$ is unnecessary for Algorithm 2 to correctly reconstruct the ARG ${𝒢}_{+}$; see also Section A.3 for the theoretical justification.

### A.3 Relaxation of Assumption 3

Assumption 3 in Theorem 4 leads to consistent identification for **V**. Now, we discuss when Algorithm 2 correctly reconstructs ${𝒢}_{+}$ without requiring Assumption 3.

**Assumption 5** *For* 1 ≤ *j* ≤ *p*, *there exists* $\tau_{j}^{*}$ *such that*

- $\left\{l:X_{l}\;\text{intervenes on}\;Y_{j}\;\text{or its unmediated parents}\right\}\subseteq \left\{l:\left|{\text{V}}_{lj}\right|\geq \tau_{j}^{*}\right\}.$
- $\tau_{j}^{*}\geq 100c_{1}^{-1}c_{2}\left({\text{Ω}}_{jj}^{-1/2}\right)\sqrt{\left(\kappa_{j}^{\circ }-\left|\left\{l:\left|{\text{V}}_{lj}\right|\geq \tau_{j}^{*}\right\}\right|+1\right)(\log(q)/n+\log(n)/n)}.$

Assumption 5 requires the effects of intervention variables on *Y_j_* or its unmediated parents to exceed a certain signal strength $\tau_{j}^{*}$, while imposing no restrictions on the other intervention variables, for 1 ≤ *j* ≤ *p*. These signals enable us to reconstruct ${𝒢}_{+}$. Assumption 3 implies Assumption 5 with $\tau_{j}^{*}=\min_{V_{lj}\neq 0}\left|{\text{V}}_{lj}\right|$, so Assumption 5 is weaker.

**Theorem 9** *Suppose Assumptions 1-2 and 5 are met with constants c*_1_ < 6*c*_2_, *and the machine precision* tol≪1/n *is negligible. For* 1 ≤ *j* ≤ *p*, *there exist some suitable choice of tuning parameters* (*κ_j_, τ_j_*) *in* Algorithm 1 *such that*

$$\left|\left\{l:\left|{\text{V}}_{lj}\right|\geq \tau_{j}^{*}\right\}\right|\leq \kappa_{j}\leq \kappa_{j}^{\circ },\;\frac{36c_{2}}{c_{1}}\sqrt{{\text{Ω}}_{jj}^{-1}\left(\frac{\log(q)}{n}+\frac{\log(n)}{n}\right)}\leq \tau_{j}\leq \frac{2\tau_{j}^{*}}{5},$$

*then for any γ_j_ such that*

$$\tau_{j}^{-1}{\left(32c_{2}^{2}{\text{Ω}}_{jj}^{-1}n^{-1}(\log(q)+\log(n))\right)}^{1/2}\leq \gamma_{j}\leq c_{1}/6\sqrt{\kappa_{j}^{\circ }-\left|\left\{l:\left|{\text{V}}_{lj}\right|\geq \tau_{j}^{*}\right\}\right|+1},$$

*almost surely we have* Algorithm 1 *terminates in at most* $1+\lceil \log\left(\kappa_{\max}^{\circ }\right)/\log(4)\rceil$ *DC iterations when n is sufficiently large. Moreover, almost surely we have* Algorithm 2 *recovers* ${ℰ}_{+}$ *and* ${ℐ}_{+}$ *when n is sufficiently large*.

The proof of Theorem 9 is given in Appendix B.9.

### A.4 Comparison of strong faithfulness and Assumption 3 (or 5)

In the literature, a faithfulness condition is usually assumed for identifiability up to Markov equivalence classes (Spirtes et al., 2000). For discussion, we formally introduce the concepts of faithfulness and strong faithfulness.

Consider a DAG 𝒢 with node variables ${\left(Z_{1},\ldots ,Z_{p+q}\right)}^{⊤}$. Nodes *Z_i_* and *Z_j_* are adjacent if $Z_{i}\to Z_{j}$ or $Z_{j}\to Z_{i}$. A *path* (*undirected*) between *Z_i_* and *Z_j_* in 𝒢 is a sequence of distinct nodes (*Z_i_*, … , *Z_j_*) such that all pairs of successive nodes in the sequence are adjacent. A nonendpoint node *Z_k_* on a path $\left(Z_{i},\ldots ,Z_{k-1},Z_{k},Z_{k+1},\ldots ,Z_{j}\right)$ is a *collider* if $Z_{k-1}\to Z_{k}\leftarrow Z_{k+1}$. Otherwise it is a *noncollider*. Let A⊆{1,…,p+q}, where *A* does not contain *i* and *j*. Then ***Z**_A_ blocks* a path $\left(Z_{i},\ldots ,Z_{j}\right)$ if at least one of the following holds: (i) the path contains a noncollider that is in ***Z****_A_*, or (ii) the path contains a collider that is not in ***Z**_A_* and has no descendant in ***Z****_A_*. A node *Z_i_* is d-separated from *Z_j_* given ***Z**_A_* if ***Z**_A_* block every path between *Z_i_* and $Z_{j};i\neq j$ (Pearl, 2009).

According to Uhler et al. (2013), a multivariate Gaussian distribution of ${\left(Z_{1},\ldots ,Z_{p+q}\right)}^{⊤}$ is said to be *ς*-strong faithful to a DAG with node set 𝒱={1,…,p+q} if

(24)
$$\underset{A\subseteq 𝒱\setminus \{i,j\}}{\min}\left\{\left|\text{Corr}\left(Z_{i},Z_{j}|Z_{A}\right)\right|:Z_{i}\;\text{is not d-separated from}\;Z_{j}\;\text{given}\;Z_{A}\right\}>\varsigma ,$$

for 1≤i≠j≤p+q, where *ς* ∈ [0, 1), Corr denotes the correlation. When *ς* = 0, (24) is equivalent to faithfulness. For consistent structure learning (up to Markov equivalence classes), it often requires that $\varsigma ≳\sqrt{s_{0}\log(p+q)/n}$, where *s*_0_ is a sparsity measure; see Uhler et al. (2013) for a survey. For a pair (*i, j*), the number of possible sets for *A* is 2^(*p*+*q*−2)^. If *Z_i_* → *Z_j_*, then $\text{Corr}\left(Z_{i},Z_{j}|Z_{A}\right)\neq 0$ for any *A*. Therefore, for this (*i, j*) pair alone, (24) could require exponentially many conditions. Indeed, (24) is very restrictive in high-dimensional situation (Uhler et al., 2013).

By comparison, Algorithm 2 yields consistent structure learning based on Assumption 3 or 5 instead of strong faithfulness. In some sense, Assumption 3 or 5 requires the sufficient signal strength that is analogous to the condition for consistent feature selection (Shen et al., 2012). This assumption may be thought of as an alternative to strong faithfulness. As illustrated in Example 4, Assumption 3 or 5 is less stringent than strong faithfulness.

**Example 4 (Faithfulness)** *Assume **X*** ~ *N*(**0, I**). *Consider* model (1) *with p* = *q* = 3,

$$Y_{1}={\text{W}}_{11}X_{1}+\varepsilon_{1},\;Y_{2}={\text{U}}_{12}Y_{1}+{\text{W}}_{22}X_{2}+\varepsilon_{2},\;Y_{3}={\text{U}}_{13}Y_{1}+{\text{U}}_{23}Y_{2}+{\text{W}}_{33}X_{3}+\varepsilon_{3},$$

*where* $\varepsilon_{1},\varepsilon_{2},\varepsilon_{3}∼N(0,1)$ *are independent and* ${\text{U}}_{12},{\text{U}}_{13},{\text{U}}_{23},{\text{W}}_{11},{\text{W}}_{22},{\text{W}}_{33}\neq 0$. *Denote* $Z={\left(Y_{1},Y_{2},Y_{3},X_{1},X_{2},X_{3}\right)}^{⊤}$. *Since the directed relations among **X** are not of interest*, (24) *becomes*

(25)
$$\underset{\begin{array}{c} Z_{A}=\left(Y_{A_{1}},X_{A_{2}}\right):\\ A_{1}\subseteq {\left\{i,j\right\}}^{c},A_{2} \end{array}}{\min}\left\{\left|\text{Corr}\left(Y_{i},Y_{j}|Z_{A}\right)\right|:Y_{i},Y_{j}\;\mathrm{are not d-separated given}\;Z_{A}\right\}>\varsigma ,\;\underset{\begin{array}{c} Z_{A}=\left(Y_{A_{1}},X_{A_{2}}\right):\\ A_{1}\subseteq {\left\{j\right\}}^{c},A_{2}\subseteq {\left\{\right\}}^{c} \end{array}}{\min}\left\{\left|\text{Corr}\left(Y_{j},X_{l}|Z_{A}\right)\right|:Y_{j},X_{l}\;\mathrm{are not d-separated given}\;Z_{A}\right\}>\varsigma ,$$

*for each pair* (*i, j*) *with i* ≠ *j and each pair* (*l, j*). *Then strong-faithfulness in* (25) *assumes* 152 *conditions for the correlations. By comparison, Assumption 3 requires the absolute values of* ${\text{V}}_{11},{\text{V}}_{12},{\text{V}}_{13},{\text{V}}_{22},{\text{V}}_{23},{\text{V}}_{33}≳\sqrt{\log(q)/n}$, *which in turn requires the minimal absolute value of six correlations* $\geq \sqrt{\log(q)/n}$,

$$\left(i\right)\text{Corr}\left(Y_{1},X_{1}|X_{2},X_{3}\right),\;\left(ii\right)\text{Corr}\left(Y_{2},X_{1}|X_{2},X_{3}\right),\;\left(iii\right)\text{Corr}\left(Y_{3},X_{1}|X_{2},X_{3}\right),\;\left(iv\right)\text{Corr}\left(Y_{2},X_{2}|X_{1},X_{3}\right),\;\left(v\right)\text{Corr}\left(Y_{3},X_{2}|X_{1},X_{3}\right),\;\left(vi\right)\text{Corr}\left(Y_{3},X_{3}|X_{1},X_{2}\right).$$

*Importantly, (i)-(vi) are required in* (25), *suggesting that the strong-faithfulness is more stringent than Assumption 3.*

### A.5 Irregular hypothesis

Assume, without loss of generality, that $\hat{𝒟}=𝒟$ and ${\hat{𝒢}}_{+}=𝒢$ are correctly reconstructed in the following discussion.

- For testing of directed edges (2), suppose *H*_0_ is irregular, namely, 𝒟∪ℰ contains a directed cycle. This implies that a directed cycle exists in $\hat{𝒟}\cup {\hat{ℰ}}_{+}$. In this situation, we decompose *H*_0_ into sub-hypotheses $H_{0}^{\left(1\right)},\ldots ,H_{0}^{\left(\nu \right)}$, each of which is regular. Then testing *H*_0_ is equivalent to multiple testing for $H_{0}^{\left(1\right)},\ldots ,H_{0}^{\left(\nu \right)}$. For instance, in Example 1, *H*_0_ : U_45_ = U_53_ = 0 is irregular, and *H*_0_ can be decomposed into $H_{0}^{\left(1\right)}:{\text{U}}_{45}=0$ and $H_{0}^{\left(2\right)}:{\text{U}}_{53}=0$.
- For testing of directed pathways (3), if *H*_0_ is irregular, then $\hat{𝒟}\cup {\hat{ℰ}}_{+}$ has a directed cycle. The p-value is defined to be one in this situation since no evidence supports the presence of the pathway.

### A.6 Theoretical results on structure learning

The regression (9) can be solved by Algorithm 1 with the input **Y**_⋅*j*_ as the response variable and the input $\left({\text{Y}}_{\cdot ,{\text{AN}}_{\hat{𝒢}+}\left(j\right)},{\text{X}}_{\cdot ,{\text{IN}}_{\hat{𝒢}+}\left(j\right)}\right)$ as the covariates for 1≤j≤p. The tuning parameters for solving (9) by Algorithm 1 are denoted by ${\left\{\left(\kappa_{j}^{\prime },\tau_{j}^{\prime }\right)\right\}}_{1\leq j\leq p}$.

Let $\bar{\kappa }=\underset{1\leq j\leq p}{\max}\left|{\mathrm{AN}}_{{𝒢}_{+}}(j)\right|+\left|{\mathrm{IN}}_{{𝒢}_{+}}(j)\right|$ and **Z** = (**Y**, **X**).

**Assumption 6** *For constants c*_3_, *c*_4_ > 0,

- $\underset{\left\{A:|A|\leq 2\bar{\kappa }\right\}}{\min}\underset{\left\{\zeta :{\left\|\zeta_{A^{c}}\right\|}_{1}\leq 3{\left\|\zeta_{A}\right\|}_{1}\right\}}{\min}{\left\|Z\zeta \right\|}_{2}^{2}/n{\left\|\zeta \right\|}_{2}^{2}\geq c_{3}.$
- $\underset{1\leq k\leq p+q}{\max}n^{-1}{\left(Z^{⊤}Z\right)}_{kk}\leq c_{4}^{2}.$

**Assumption 7** $\underset{{\text{U}}_{kj}\neq 0}{\min}\left|{\text{U}}_{kj}\right|\geq 100c_{3}^{-1}c_{4}\underset{1\leq j\leq p}{\max}\left(\sigma_{j}\right)\sqrt{\log(p)/n+\log(n)/n}$.

**Theorem 10** *Suppose the assumptions in Theorem 4 are satisfied. In addition, suppose Assumptions 6-7 are met with constants c*_3_ < 6*c*_4_. *For* 1≤j≤p, *assuming* $\left|{\mathrm{AN}}_{𝒢}(j)\right|\ll n$ *and* $\left|{\mathrm{IN}}_{𝒢}(j)\right|\ll n$, *if the tuning parameters* $\left(\kappa_{j}^{\prime },\tau_{j}^{\prime }\right)$ *are suitably chosen such that*

$$\kappa_{j}^{\prime }=\left|{\text{PA}}_{𝒢}(j)\right|,\;\frac{36c_{4}}{c_{3}}\sigma_{j}\sqrt{\frac{\log(p)}{n}+\frac{\log(n)}{n}}\leq \tau_{j}^{\prime }\leq \frac{2}{5}\underset{{\text{U}}_{kj}\neq 0}{\min}\left|{\text{U}}_{kj}\right|,$$

*then for any γ_j_ such that* ${\left(\tau_{j}^{\prime }\right)}^{-1}{\left(32c_{4}^{2}\sigma_{j}^{2}n^{-1}(\log(p)+\log(n))\right)}^{1/2}\leq \gamma_{j}\leq c_{3}/6$, *almost surely we have* $\hat{ℰ}=ℰ$, *when n is sufficiently large.*

## Appendix B. Technical proofs

### B.1 Proof of Proposition 1

Suppose that ***θ*** = (**U, W, Σ**) and $\tilde{\theta }=(\tilde{U},\tilde{W},\tilde{\Sigma })$ render the same distribution of (***Y***, ***X***). We will prove that $\theta =\tilde{\theta }$.

Denote by 𝒢(θ) and $𝒢(\tilde{\theta })$ the DAGs corresponding to ***θ*** and $\tilde{\theta }$, respectively. First, consider 𝒢(θ). Without loss of generality, assume *Y*_1_ is a leaf node in 𝒢(θ). By Assumption 1C, there exists an instrumental intervention with respect to 𝒢(θ), say *X*_1_. Then,

(26)
$$\mathrm{Cov}\left(Y_{j},X_{1}|X_{\left\{2,\ldots ,q\right\}}\right)=0,\;j=2,\ldots ,p,$$

(27)
$$\mathrm{Cov}\left(Y_{1},X_{1}|Y_{A},X_{\left\{2,\ldots ,q\right\}}\right)\neq 0,\;\text{for any}\;A\subseteq \{2,\ldots ,p\}.$$

By the local Markov property (Spirtes et al., 2000), (27) implies that $X_{1}\to Y_{1}$ in $𝒢(\tilde{\theta })$. Suppose *Y*_1_ is not a leaf node in $𝒢(\tilde{\theta })$. Without loss of generality, assume that *Y*_1_ is an unmediated parent of *Y*_2_. Then Cov(*Y*_2_, *X*_1_ | ***X***_{_2, … ,*q*_}_) = 0 but *X*_1_ → *Y*_1_ and *Y*_1_ is an unmediated parent of *Y*_2_, which contradicts to Assumption 1B. This implies that if *Y*_1_ is a leaf node in 𝒢(θ) then it must be a leaf node in $𝒢(\tilde{\theta })$. In both *G*(***θ***) and $𝒢(\tilde{\theta })$, the parents and interventions of *Y*_1_ can be identified by

$$𝔼\left(Y_{1}|Y_{\left\{2,\ldots ,p\right\}},X\right)=𝔼\left(Y_{1}|Y_{{\text{PA}}_{𝒢(\theta )}(1)},X\right)=𝔼\left(Y_{1}|Y_{{\text{PA}}_{𝒢(\theta )}(1)},X_{{\text{IN}}_{𝒢(\theta )}(1)}\right),$$

$$𝔼\left(Y_{1}|Y_{\left\{2,\ldots ,p\right\}},X\right)=𝔼\left(Y_{1}|Y_{{\text{PA}}_{𝒢(\tilde{\theta })}(1)},X\right)=𝔼\left(Y_{1}|Y_{{\text{PA}}_{𝒢(\tilde{\theta })}(1)},X_{{\text{IN}}_{𝒢(\tilde{\theta })}(1)}\right).$$

Consequently, *Y*_1_ has the same parents and interventions in 𝒢(θ) and $𝒢(\tilde{\theta })$.

The forgoing argument is applied to other nodes sequentially. First, we remove *Y*_1_ with any directed edges to *Y*_1_, which does not alter the joint distribution of (***Y***_{_2, … , p_}_, ***X***) and the sub-DAG of nodes *Y*_2_, … , *Y_p_*. By induction, we remove the leafs in 𝒢(θ) until it is empty, leading to $𝒢(\theta )=𝒢(\tilde{\theta })$. Finally, $\theta =\tilde{\theta }$ because they have the same locations for nonzero elements and these model parameters (or regression coefficients) are uniquely determined under Assumption 1A (Shojaie and Michailidis, 2010). This completes the proof.

### B.2 Proof of Proposition 2

Proof of (A)

Note that the maximal length of a path in a DAG of *p* nodes is at most *p* − 1. Then it can be verified that **U** is nilpotent in that $U^{p}=0$. An application of the matrix series expansion yields that ${\left(\text{I}-\text{U}\right)}^{-1}=\text{I}+U+\cdots +{\text{U}}^{p-1}$. Using the fact that $V=W{\left(I-U\right)}^{-1}$ from (6), we have, for any 1≤l, j≤p,

$${\text{V}}_{lj}=\sum_{k=1}^{p}\;{\text{W}}_{lk}\left({\text{I}}_{kj}+{\text{U}}_{kj}+\cdots +{\left(U^{p-1}\right)}_{kj}\right),$$

where U*_kj_* is the (*k, j*)th entry of **U**. If ${\text{V}}_{lj}\neq 0$, then there exists *k* such that $W_{lk}\neq 0$ and ${\left(U^{r}\right)}_{kj}\neq 0$ for some 0≤r≤p−1. If *r* = 0, then we must have *k* = *j*, and $X_{l}\to Y_{j}$. If *r* > 0, then $X_{l}\to Y_{k}$ and *Y_k_* is an ancestor of *Y_j_*.

Proof of (B)

First, for any leaf node variable *Y_j_*, by Assumption 1, there exists an instrument $X_{l}\to Y_{j}$. If ${\text{V}}_{lj^{\prime }}\neq 0$ for some $j^{\prime }\neq j$, then *Y_j_* must be an ancestor of *Y_j′_*, which contradicts the fact that *Y_j_* is a leaf node variable.

Conversely, suppose that ${\text{V}}_{lj}\neq 0$ and ${\text{V}}_{lj^{\prime }}=0$ for $j^{\prime }\neq j$. If *Y_j_* is not a leaf node variable, then there exists a variable $Y_{j^{\prime }}$ such that *Y_j_* is an unmediated parent of $Y_{j^{\prime }}$, that is ${\text{U}}_{jj^{\prime }}\neq 0$ and ${\left(U^{r}\right)}_{jj^{\prime }}=0$ for *r* > 1. Then ${\text{V}}_{lj^{\prime }}={\text{W}}_{lj}{\text{U}}_{jj^{\prime }}\neq 0$, a contradiction.

### B.3 Proof of Proposition 3

Suppose *Y_k_* is an unmediated parent of *Y_j_*. Let *X_l_* be an instrument of *Y_k_* in ${𝒢}_{{ℒ}^{c}}$. Then there are two cases: (1) *X_l_* intervenes on *Y_k_* but does not intervene on *Y_j_*, namely *X_l_* → *Y_k_* but $X_{l}↛Y_{j}$; (2) *X_l_* intervenes on *Y_k_* and *Y_j_* simultaneously, namely $X_{l}\to Y_{k}$ and $X_{l}\to Y_{j}$. For (1), ${\text{V}}_{lj}={\text{W}}_{lk}{\text{U}}_{kj}\neq 0$. For (2), Assumption 1B implies that ${\text{V}}_{lj}\neq 0$. This holds for every instrument of *Y_k_* in ${𝒢}_{{ℒ}^{c}}$, and the desired result follows.

Conversely, suppose for each instrument *X_l_* of *Y_k_* in ${𝒢}_{{ℒ}^{c}}$, we have ${\text{V}}_{lj}\neq 0$. Let *X*_*l*′_ be an instrument of *Y_k_* in 𝒢, which is also an instrument in ${𝒢}_{{ℒ}^{c}}$. Then $0\neq {\text{V}}_{l^{\prime }j}={\text{W}}_{l^{\prime }k}{\text{U}}_{kj}$, which implies ${\text{U}}_{kj}\neq 0$. This completes the proof.

### B.4 Proof of Theorem 4

The proof proceeds in two steps: (A) we show that $\left\{l:{\hat{\text{V}}}_{lj}\neq 0\right\}=\left\{l:{\text{V}}_{lj}\neq 0\right\}$ almost surely for 1≤j≤p; and (B) we show that ${\hat{𝒢}}_{+}={𝒢}_{+}$ if $\hat{V}$ satisfies the property in (A).

Proof of (A)

Let $A_{j}^{{}^{\circ}}=\left\{l:{\text{V}}_{lj}\neq 0\right\}$ and $A_{j}^{\left[t\right]}=\left\{l:\left|{\tilde{V}}_{lj}^{\left[t\right]}\right|\geq \tau_{j}\right\}$ be the estimated support of penalized solution at the *t*-th iteration of Algorithm 1. For the penalized solution, define the false negative set ${\text{FN}}_{j}^{\left[t\right]}=A_{j}^{{}^{\circ}}\backslash A_{j}^{\left[t\right]}$ and the false positive set ${\text{FP}}_{j}^{\left[t\right]}=A_{j}^{\left[t\right]}\backslash A_{j}^{{}^{\circ}}$ for t≥0. Consider a “good” event

$${ℰ}_{j}=\left\{{\left\|X^{⊤}{\hat{\xi }}_{j}/n\right\|}_{\infty }\leq 0.5\gamma_{j}\tau_{j}\right\}\cap \left\{{\left\|{\hat{V}}_{\cdot j}^{{}^{\circ}}-V_{\cdot j}\right\|}_{\infty }\leq 0.5\tau_{j}\right\},$$

where ${\hat{\xi }}_{j}=Y_{j}-X{\hat{V}}_{\cdot j}^{{}^{\circ}}$ is the residual of the oracle least squares estimate ${\hat{\text{V}}}_{\cdot j}^{{}^{\circ}}$ such that $A_{j}=\left\{l:{\hat{\text{V}}}_{lj}^{{}^{\circ}}\neq 0\right\};1\leq j\leq p$. We shall show that ${\text{FN}}_{j}^{\left[t\right]}$ and ${\text{FP}}_{j}^{\left[t\right]}$ are eventually empty on event ${ℰ}_{j}$ which has a probability tending to one.

First, we will show that if $\left|A_{j}^{{}^{\circ}}\cup A_{j}^{\left[t-1\right]}\right|\leq 2\kappa_{\max}^{\circ }$ on ${ℰ}_{j}$ for t≥1, then $\left|A_{j}^{{}^{\circ}}\cup A_{j}^{\left[t\right]}\right|\leq 2\kappa_{\max}^{\circ }$, to be used in Assumption 2A. To proceed, suppose $\left|A_{j}^{{}^{\circ}}\cup A_{j}^{\left[t-1\right]}\right|\leq 2\kappa_{\max}^{\circ }$ on ${ℰ}_{j}$ for *t* ≥ 1. By the optimality condition of (8),

$${\left({\hat{V}}_{.j}^{{}^{\circ}}-{\tilde{V}}_{:j}^{\left[t\right]}\right)}^{⊤}\left(-X^{⊤}\left(Y_{j}-X{\tilde{V}}_{.j}^{\left[t\right]}\right)/n+\gamma_{j}\tau_{j}\nabla {\left\|{\tilde{V}}_{{\left(A_{j}^{\left[t-1\right]}\right)}^{c},j}\right\|}_{1}\right)\geq 0,$$

where ${\tilde{V}}^{\left[t\right]}$ is defined in (8). Plugging ${\hat{\xi }}_{j}=Y_{j}-X{\hat{V}}_{\cdot j}^{{}^{\circ}}$ into the inequality and rearranging it, we have ${\left\|X\left({\tilde{V}}_{\cdot j}^{\left[t\right]}-{\hat{V}}_{\cdot j}^{{}^{\circ}}\right)\right\|}_{2}^{2}/n$ is no greater than

(28)
$$\;{\left({\tilde{V}}_{\cdot j}^{\left[t\right]}-{\hat{V}}_{\cdot j}^{{}^{\circ}}\right)}^{⊤}\left(X^{⊤}{\hat{\xi }}_{j}/n-\gamma_{j}\tau_{j}\nabla {\left\|{\tilde{V}}_{{\left(A_{j}^{\left[t-1\right]}\right)}^{c},j}^{\left[t\right]}\right\|}_{1}\right)\;={\left({\tilde{V}}_{A_{j}^{{}^{\circ}}\backslash A_{j}^{\left[t-1\right]},j}^{\left[t\right]}-{\hat{V}}_{A_{j}^{{}^{\circ}}\backslash A_{j}^{\left[t-1\right]},j}^{{}^{\circ}}\right)}^{⊤}\left(X_{A_{j}^{{}^{\circ}}\backslash A_{j}^{\left[t-1\right]}}^{⊤}{\hat{\xi }}_{j}/n-\gamma_{j}\tau_{j}\nabla {\left\|{\tilde{V}}_{A_{j}^{{}^{\circ}}\backslash A_{j}^{\left[t-1\right]},j}^{\left[t\right]}\right\|}_{1}\right)\;\;+{\left({\tilde{V}}_{{\left(A_{j}^{{}^{\circ}}\cup A_{j}^{\left[t-1\right]}\right)}^{c},j}^{\left[t\right]}-{\hat{V}}_{{\left(A_{j}^{{}^{\circ}}\cup A_{j}^{\left[t-1\right]}\right)}^{c},j}^{{}^{\circ}}\right)}^{⊤}\left(X^{⊤}\hat{\xi }/n-\gamma_{j}\tau_{j}\nabla {\left\|{\tilde{V}}_{{\left(A_{j}^{{}^{\circ}}\cup A_{j}^{\left[t-1\right]}\right)}^{c},j}^{\left[t\right]}\right\|}_{1}\right)\;\;+{\left({\tilde{V}}_{A_{j}^{\left[t-1\right]}\backslash A_{j}^{{}^{\circ}},j}^{\left[t\right]}-{\hat{V}}_{A_{j}^{\left[t-1\right]}\backslash A_{j}^{{}^{\circ}},j}^{{}^{\circ}}\right)}^{⊤}X_{A_{j}^{\left[t-1\right]}\backslash A_{j}^{{}^{\circ}}}^{⊤}\hat{\xi }/n,$$

where $X_{A_{j}^{*}}^{⊤}{\hat{\xi }}_{j}/n=0$ has been used. Note that

$${\left({\tilde{V}}_{{\left(A_{j}^{{}^{\circ}}\cup A_{j}^{\left[t-1\right]}\right)}^{c},j}^{\left[t\right]}-{\hat{V}}_{{\left(A_{j}^{{}^{\circ}}\cup A_{j}^{\left[t-1\right]}\right)}^{c},j}^{{}^{\circ}}\right)}^{⊤}\nabla {\left\|{\tilde{V}}_{{\left(A_{j}^{{}^{\circ}}\cup A_{j}^{\left[t-1\right]}\right)}^{c},j}^{[t}\right\|}_{1}={\left\|{\tilde{V}}_{{\left(A_{j}^{{}^{\circ}}\cup A_{j}^{\left[t-1\right]}\right)}^{c},j}^{\left[t\right]}-{\hat{V}}_{{\left(A_{j}^{{}^{\circ}}\cup A_{j}^{\left[t-1\right]}\right)}^{c},j}^{{}^{\circ}}\right\|}_{1}.$$

Then (28) is no greater than

(29)
$${\left\|{\tilde{V}}_{A_{j}^{{}^{\circ}}\text{Δ}A_{j}^{\left[t-1\right]},j}^{\left[t\right]}-{\hat{V}}_{A_{j}^{{}^{\circ}}\text{Δ}A_{j}^{\left[t-1\right]},j}^{{}^{\circ}}\right\|}_{1}\left({\left\|X^{⊤}{\hat{\xi }}_{j}/n\right\|}_{\infty }+\gamma_{j}\tau_{j}\right)\;+{\left\|{\tilde{V}}_{{\left(A_{j}^{{}^{\circ}}\cup A_{j}^{\left[t-1\right]}\right)}^{c},j}^{\left[t\right]}-{\hat{V}}_{{\left(A_{j}^{{}^{\circ}}\cup A_{j}^{\left[t-1\right]}\right)}^{c},j}^{{}^{\circ}}\right\|}_{1}\left({\left\|X^{⊤}{\hat{\xi }}_{j}/n\right\|}_{\infty }-\gamma_{j}\tau_{j}\right),$$

where Δ denotes the symmetric difference of two sets. Note that ${\left\|X\left({\tilde{V}}_{\cdot j}^{\left[t\right]}-{\hat{V}}_{\cdot j}^{{}^{\circ}}\right)\right\|}_{2}^{2}/n\geq 0$. Rearranging the inequality yields that

$$\left(\gamma_{j}\tau_{j}-{\left\|X^{⊤}{\hat{\xi }}_{j}/n\right\|}_{\infty }\right){\left\|{\tilde{V}}_{{\left(A_{j}^{{}^{\circ}}\cup A_{j}^{\left[t-1\right]}\right)}^{c},j}^{\left[t\right]}-{\hat{V}}_{{\left(A_{j}^{{}^{\circ}}\cup A_{j}^{\left[t-1\right]}\right)}^{c},j}^{{}^{\circ}}\right\|}_{1}\;\leq \left({\left\|X^{⊤}{\hat{\xi }}_{j}/n\right\|}_{\infty }+\gamma_{j}\tau_{j}\right){\left\|{\tilde{V}}_{A_{j}^{{}^{\circ}}\text{Δ}A_{j}^{\left[t]-1\right]},j}^{\left[t\right]}-{\hat{V}}_{A_{j}^{{}^{\circ}}\text{Δ}A_{j}^{\left[t-1\right]},j}^{{}^{\circ}}\right\|}_{1}.$$

On event ${ℰ}_{j}$, ${\left\|X^{⊤}{\hat{\xi }}_{j}/n\right\|}_{\infty }\leq \gamma_{j}\tau_{j}/2$, implying that

$${\left\|{\tilde{V}}_{{\left(A_{j}^{{}^{\circ}}\cup A_{j}^{\left[t-1\right]}\right)}^{c},j}^{\left[t\right]}-{\hat{V}}_{{\left(A_{j}^{{}^{\circ}}\cup A_{j}^{\left[t-1\right]}\right)}^{c},j}^{{}^{\circ}}\right\|}_{1}\leq 3{\left\|{\tilde{V}}_{A_{j}^{{}^{\circ}}\text{Δ}A_{j}^{\left[t-1\right]},j}^{\left[t\right]}-{\hat{V}}_{A_{j}^{{}^{\circ}}\text{Δ}A_{j}^{\left[t-1\right]},j}^{{}^{\circ}}\right\|}_{1}\;\;\;\;\;\;\;\;\;\;\;\;\;\;\;\leq 3{\left\|{\tilde{V}}_{A_{j}^{{}^{\circ}}\cup A_{j}^{\left[t-1\right]},j}^{\left[t\right]}-{\hat{V}}_{A_{j}^{{}^{\circ}}\cup A_{j}^{\left[t-1\right]},j}^{{}^{\circ}}\right\|}_{1}.$$

Note that $\left|A_{j}^{{}^{\circ}}\cup A_{j}^{\left[t-1\right]}\right|\leq 2\kappa_{\max^{{}^{\circ}}}$. By Assumption 2A and (29),

(30)
$$c_{1}{\left\|{\tilde{V}}_{\cdot j}^{\left[t\right]}-{\hat{V}}_{\cdot j}^{{}^{\circ}}\right\|}_{2}^{2}\leq \left({\left\|X^{⊤}{\hat{\xi }}_{j}/n\right\|}_{\infty }+\gamma_{j}\tau_{j}\right){\left\|{\tilde{V}}_{A_{j}^{{}^{\circ}}△A_{j}^{\left[t-1\right]},j}^{\left[t\right]}-{\hat{V}}_{A_{j}^{{}^{\circ}}△A_{j}^{\left[t-1\right]},j}^{{}^{\circ}}\right\|}_{1}\;\;\;\;\;\;\;\;\;+\left({\left\|X^{⊤}{\hat{\xi }}_{j}/n\right\|}_{\infty }-\gamma_{j}\tau_{j}\right){\left\|{\tilde{V}}_{{\left(A_{j}^{{}^{\circ}}\cup A_{j}^{\left[t-1\right]}\right)}^{c},j}^{\left[t\right]}-{\hat{V}}_{{\left(A_{j}^{{}^{\circ}}\cup A_{j}^{\left[t-1\right]}\right)}^{c},j}^{{}^{\circ}}\right\|}_{1}\;\;\;\;\;\;\;\leq \left({\left\|X^{⊤}{\hat{\xi }}_{j}/n\right\|}_{\infty }+\gamma_{j}\tau_{j}\right){\left\|{\tilde{V}}_{A_{j}^{{}^{\circ}}\text{Δ}A_{j}^{\left[t-1\right]},j}^{\left[t\right]}-{\hat{V}}_{A_{j}^{{}^{\circ}}\text{Δ}A_{j}^{\left[t-1\right]},j}^{{}^{\circ}}\right\|}_{1}.$$

By the Cauchy-Schwarz inequality,

$${\left\|{\tilde{V}}_{A_{j}^{{}^{\circ}}\text{Δ}A_{j}^{\left[t]-1\right]},j}^{\left[t\right]}-{\hat{V}}_{A_{j}^{{}^{\circ}}\text{Δ}A_{j}^{\left[t-1\right]},j}^{{}^{\circ}}\right\|}_{1}\leq \sqrt{A_{j}^{{}^{\circ}}\text{Δ}A_{j}^{\left[t-1\right]}}{\left\|{\tilde{V}}_{\cdot j}^{\left[t\right]}-{\hat{V}}_{\cdot j}^{{}^{\circ}}\right\|}_{2}.$$

Thus, $c_{1}{\left\|{\tilde{V}}_{\cdot j}^{\left[t\right]}-{\hat{V}}_{\cdot j}^{{}^{\circ}}\right\|}_{2}\leq 1.5\gamma_{j}\tau_{j}\sqrt{2\kappa_{\max}^{\circ }}$ since $\left|A_{j}^{{}^{\circ}}\text{Δ}A_{j}^{\left[t-1\right]}\right|\leq \left|A_{j}^{{}^{\circ}}\cup A_{j}^{\left[t-1\right]}\right|\leq 2\kappa_{\max}^{{}^{\circ}}$ and ${\left\|X^{⊤}{\hat{\xi }}_{j}/n\right\|}_{\infty }\leq 0.5\gamma_{j}\tau_{j}$. By the condition of Theorem 4, ${\left\|{\tilde{V}}_{\cdot j}^{\left[t\right]}-{\hat{V}}_{\cdot j}^{{}^{\circ}}\right\|}_{2}/\tau_{j}\leq \sqrt{\kappa_{\max}^{\circ }}$ and $\gamma_{j}\leq c_{1}/6$. On the other hand, for any $l\in {\text{FP}}_{j}^{\left[t\right]}=A_{j}^{\left[t\right]}\backslash A_{j}^{{}^{\circ}}$, we have $\left|{\tilde{\text{V}}}_{lj}^{\left[t\right]}-{\hat{\text{V}}}_{lj}^{{}^{\circ}}\right|=\left|{\tilde{\text{V}}}_{lj}^{\left[t\right]}\right|>\tau_{j}$. Thus, $\sqrt{\left|{\text{FP}}_{j}^{\left[t\right]}\right|}\leq {\left\|{\tilde{V}}_{.j}^{\left[t\right]}-{\hat{V}}_{\cdot j}^{{}^{\circ}}\right\|}_{2}/\tau_{j}\leq \sqrt{\kappa_{\max}^{\circ }}$, implying $\left|A_{j}^{{}^{\circ}}\cup A_{j}^{\left[t\right]}\right|=\left|A_{j}^{{}^{\circ}}\right|+\left|{\text{FP}}_{j}^{\left[t\right]}\right|\leq 2\kappa_{\max}^{\circ }$ on ${ℰ}_{j}$ for t≥1.

Next, we estimate the number of iterations required for termination. Note that the machine precision is negligible. The termination criterion is met when $A_{j}^{\left[t\right]}=A_{j}^{\left[t-1\right]}$ since the weighted Lasso problem (8) remains same at the *t*th and (*t* − 1)th iterations. To show that $A_{j}^{\left[t\right]}=A_{j}^{{}^{\circ}}$ eventually, we prove that $\left|{\text{FN}}_{j}^{\left[t\right]}\right|+\left|{\text{FP}}_{j}^{\left[t\right]}\right|<1$ eventually.

Now, suppose $\left|{\text{FN}}_{j}^{\left[t\right]}\right|+\left|{\text{FP}}_{j}^{\left[t\right]}\right|\geq 1$. For any $l\in {\text{FN}}_{j}^{\left[t\right]}\cup {\text{FP}}_{j}^{\left[t\right]}$, by Assumption 3,

$$\left|{\tilde{\text{V}}}_{lj}^{\left[t\right]}-{\hat{\text{V}}}_{lj}^{{}^{\circ}}\right|\geq \left|{\tilde{\text{V}}}_{lj}^{\left[t\right]}-{\text{V}}_{lj}\right|-\left|{\hat{\text{V}}}_{lj}^{{}^{\circ}}-{\text{V}}_{lj}\right|\geq \tau_{j}-0.5\tau_{j},$$

so $\sqrt{\left|{\text{FN}}_{j}^{\left[t\right]}\right|+\left|{\text{FP}}_{j}^{\left[t\right]}\right|}\leq {\left\|{\tilde{\text{V}}}_{\cdot j}^{\left[t\right]}-{\hat{V}}_{\cdot j}^{{}^{\circ}}\right\|}_{2}/0.5\tau_{j}$. By (30) and the Cauchy-Schwarz inequality, $c_{1}{\left\|{\tilde{V}}_{\cdot j}^{\left[t\right]}-{\hat{V}}_{\cdot j}^{{}^{\circ}}\right\|}_{2}\leq 1.5\gamma_{j}\tau_{j}\sqrt{\left|{\text{FN}}_{j}^{\left[t-1\right]}\right|+\left|{\text{FP}}_{j}^{\left[t-1\right]}\right|}$. By conditions (1) and (2) for (*τ_j_*, *γ_j_*) in Theorem 4, we have

$$\sqrt{\left|{\text{FN}}_{j}^{\left[t\right]}\right|+\left|{\text{FP}}_{j}^{\left[t\right]}\right|}\leq \frac{{\left\|{\tilde{V}}_{\cdot j}^{\left[t\right]}-{\hat{V}}_{\cdot j}^{{}^{\circ}}\right\|}_{2}}{0.5\tau_{j}}\leq \frac{3\gamma_{j}}{c_{1}}\sqrt{\left|{\text{FN}}_{j}^{\left[t-1\right]}\right|+\left|{\text{FP}}_{j}^{\left[t-1\right]}\right|}\;\;\;\;\;\;\;\;\;\;\;\;\;\;\leq 0.5\sqrt{\left|{\text{FN}}_{j}^{\left[t-1\right]}\right|+\left|{\text{FP}}_{j}^{\left[t-1\right]}\right|}.$$

Hence, $\sqrt{\left|{\text{FN}}_{j}^{\left[t\right]}\right|+\left|{\text{FP}}_{j}^{\left[t\right]}\right|}\leq {\left(1/2\right)}^{t}\sqrt{\left|A_{j}^{{}^{\circ}}\right|+\left|A_{j}^{\left[0\right]}\right|}$. In particular, for $t\geq 1+\left\lceil \log\kappa_{j}^{{}^{\circ}}/\log4\right\rceil$, $\left|{\text{FN}}_{j}^{\left[t\right]}\right|+\left|{\text{FP}}_{j}^{\left[t\right]}\right|<1$ implying that ${\text{FN}}_{j}^{\left[t\right]}=\emptyset$ and ${\text{FP}}_{j}^{\left[t\right]}=\emptyset$.

Let $t_{\max}=1+\left\lceil \log\kappa_{j}^{{}^{\circ}}/\log4\right\rceil$. Then ${\text{FN}}_{j}^{\left[t_{\max}\right]}={\text{FP}}_{j}^{\left[t_{\max}\right]}=\emptyset$ on event ${ℰ}_{j}$. By the condition of Theorem 4, we have $\left\{l:\;{\tilde{\text{V}}}_{lj}^{\left[t_{\max}\right]}\neq 0\right\}=\left\{l:{\text{V}}_{lj}\neq 0\right\}$. To bound $\mathbb{P}\left(\cup_{j=1}^{p}{ℰ}_{j}^{c}\right)$, let $\eta =X^{⊤}\left(I-P_{A_{j}^{{}^{\circ}}}\right)\xi_{j}$ and $\eta^{\prime }=n{\left(X_{A_{j}^{{}^{\circ}}}^{⊤}X_{A_{j}^{{}^{\circ}}}\right)}^{-1}X_{A_{j}^{{}^{\circ}}}^{⊤}\xi_{j}$, where $\xi_{j}={\left({\left(\varepsilon_{V}\right)}_{1j},\ldots ,{\left(\varepsilon_{V}\right)}_{nj}\right)}^{⊤}$. Then $\eta \in {\mathbb{R}}^{q}$ and $\eta^{\prime }\in {\mathbb{R}}^{\left|\kappa_{j}^{{}^{\circ}}\right|}$ are Gaussian vectors with $\mathrm{Var}\left(\eta_{l}\right)\leq {\text{Ω}}_{jj}^{-1}{\left(X^{⊤}X\right)}_{ll}\leq n{\text{Ω}}_{jj}^{-1}c_{2}^{2}$ and $\mathrm{Var}\left(\eta_{l}^{\prime }\right)\leq {\text{Ω}}_{jj}^{-1}{\left({\left(X_{A_{j}^{{}^{\circ}}}^{⊤}X_{A_{j}^{{}^{\circ}}}\right)}^{-1}\right)}_{ll}\leq n{\text{Ω}}_{jj}^{-1}c_{2}^{2}$. Then

$$\mathbb{P}\left({\left\|X^{⊤}{\hat{\xi }}_{j}/n\right\|}_{\infty }>0.5\gamma_{j}\tau_{j}\right)=\mathbb{P}\left({\left\|\eta /n\right\|}_{\infty }>0.5\gamma_{j}\tau_{j}\right)\;\;\;\;\;\;\;\;\;\;\;\;\;\leq \sum_{l=1}^{q}\mathbb{P}\left(\left|\eta_{l}\right|\geq 0.5n\gamma_{j}\tau_{j}\right)\;\;\;\;\;\;\;\;\;\;\;\;\;\leq q\int_{\frac{0.5\sqrt{n{\text{Ω}}_{jj}}\gamma_{j}\tau_{j}}{c_{2}}}^{\infty }\frac{e^{-t^{2}/2}}{\sqrt{2\pi }}dt\leq q\sqrt{\frac{2}{\pi }}\exp\left(-\frac{n{\text{Ω}}_{jj}\gamma_{j}^{2}\tau_{j}^{2}}{8c_{2}^{2}}\right),$$

and similarly,

$$\mathbb{P}\left({\left\|{\hat{V}}_{\cdot j}^{{}^{\circ}}-{\text{V}}_{\cdot j}^{{}^{\circ}}\right\|}_{\infty }>0.5\tau_{j}\right)\leq \sum_{l\in A_{j}^{{}^{\circ}}}\mathbb{P}\left(\left|\eta_{l}^{\prime }\right|>0.5\tau_{j}\right)\leq \kappa_{\max}^{\circ }\sqrt{\frac{2}{\pi }}\exp\left(-\frac{n{\text{Ω}}_{jj}\tau_{j}^{2}}{8c_{2}^{2}}\right).$$

Under conditions for (*τ_j_*, *γ_j_*) in Theorem 4,

$$\mathbb{P}\left(\left\{l:{\tilde{\text{V}}}_{lj}^{\left[t_{\max}\right]}\neq 0\right\}=\left\{l:{\text{V}}_{lj}\neq 0\right\};1\leq j\leq p\right)\;\geq 1-\mathbb{P}\left(\overset{p}{\underset{j=1}{\cup }}{ℰ}_{j}^{c}\right)\;\geq 1-p\sqrt{\frac{2}{\pi }}\left(e^{-4(\log(q)+\log(n))+\log(q)}+e^{-144c_{1}^{-2}c_{2}^{2}(\log(q)+\log(n))+\log\left(\kappa_{j}^{{}^{\circ}}\right)}\right)\geq 1-\sqrt{\frac{2}{\pi }}pq^{-3}n^{-4},$$

where *c*_1_ < 6*c*_2_ is used in the last inequality. By the Borel-Cantelli lemma, $\left\{l:\;{\tilde{\text{V}}}_{lj}^{\left[t_{\max}\right]}\neq 0\right\}=\left\{l:{\text{V}}_{lj}\neq 0\right\};1\leq j\leq p$ almost surely when *n* is sufficiently large.

So far, we have ${\tilde{V}}_{\cdot j}={\tilde{V}}_{\cdot j}^{\left[t_{\max}\right]}={\hat{V}}_{\cdot j}^{{}^{\circ}}$; 1≤j≤p, almost surely. It remains to show that ${\hat{\text{V}}}_{\cdot j}^{{}^{\circ}}$ is a global minimizer of (7); 1≤j≤p, with a high probability. Note that Assumptions 2A and 3 imply the degree of separation condition (Shen et al., 2013), By Theorem 2 of Shen et al. (2013),

$$\mathbb{P}\left({\hat{V}}_{\cdot j}^{{}^{\circ}}\;\text{is not a global minimizer of}\;(7);1\leq j\leq p\right)\leq 3p\exp(-2(\log(q)+\log(n))).$$

implying that almost surely ${\hat{V}}_{\cdot j}={\tilde{V}}_{\cdot j}^{\left[t_{\max}\right]}={\hat{V}}_{\cdot j}^{{}^{\circ}}$ is a global minimizer of (7) when *n* is sufficiently large. This completes the proof.

Proof of (B)

Assume $\hat{\text{V}}$ satisfies the properties in (A). Then Propositions 2 and 3 holds true for $\hat{\text{V}}$. Clearly, we have ${\hat{ℐ}}_{+}:=\{(l,j):{\hat{\text{V}}}_{lk}\neq 0$ if *k* = *j* or $(k,j)\in {\hat{ℰ}}_{+}\}={ℐ}_{+}$ whenever ${\hat{ℰ}}_{+}={ℰ}_{+}$. Thus, we only need to show ${\hat{ℰ}}_{+}={ℰ}_{+}$. We shall prove this by induction.

Given ${𝒢}^{\text{work}}$ and **V**^work^, the set of instruments on leaves is

$$ℬ=\left\{l:l\;\text{minimizes}\;{\left\|{\hat{V}}_{l,\cdot }^{\text{work}}\right\|}_{0}\;\text{and}\;{\left\|{\hat{V}}_{l,\cdot }^{\text{work}}\right\|}_{0}>0\right\}=\left\{l:{\left\|V_{l,\cdot }^{\text{work}}\right\|}_{0}=1\right\}.$$

Hence, *X_l_* is an instrument of leaf variable *Y_k_* in ${𝒢}^{\text{work}}$, when l∈ℬ and *k* maximizes $\left|V_{lk}^{work}\right|$. Hence, ℒ is the index set of leaves in ${𝒢}^{\text{work}}$. By Proposition 3, all (*k*, *j*) such that *Y*_*k*_ is an unmediated parent of a peeled variable *Y_j_* are in ${\hat{ℰ}}_{+}$ and can be identified by Assumption 1B. In model (1), after removing $Y_{ℒ}$, the local Markov property of the rest variables in ${𝒢}^{\text{work}}$ remain intact. Then we repeat the procedure until all primary variables are removed.

As a result, Algorithm 2 correctly identifies a subset of ${ℰ}_{+}$ that contains all edges from an unmediated parent, so it suffices to recover ${ℰ}_{+}$. Consequently, ${ℰ}_{+}$ can be reconstructed by Step 9 and ${ℐ}_{+}$ can be recovered by Step 10. This completes the proof of (B).

**Lemma 11** *Let*

(31)
$$T_{j}=\frac{e_{j}^{⊤}\left(P_{A_{j}}-P_{B_{j}}\right)e_{j}}{e_{j}^{⊤}\left(I-P_{A_{j}}\right)e_{j}/\left(n-\left|A_{j}\right|\right)},\;T_{j}^{*}=\frac{{\left(e_{j}^{*}\right)}^{⊤}\left(P_{A_{j}}^{*}-P_{B_{j}}^{*}\right)e_{j}^{*}}{{\left(e_{j}^{*}\right)}^{⊤}\left(I-P_{A_{j}}^{*}\right)e_{j}^{*}/\left(n-\left|A_{j}\right|\right)},$$

*where* $A_{j}={\mathrm{PA}}_{𝒮}(j)\cup {\text{IN}}_{𝒮}(j)$ *and* $B_{j}^{{}^{\circ}}=\left({\text{PA}}_{𝒮}(j)\cup {\text{IN}}_{𝒮}(j)\right)\backslash {\text{D}}_{𝒮}(j)$. *Then* (*T*_1_, … ,*T_p_*) *are independent and* $\left(T_{1}^{*},\ldots ,T_{p}^{*}\right)$ *are conditionally independent given* **Z**. *Moreover*,

$$T_{j}^{*}/\left(\left|A_{j}\right|-\left|B_{j}\right|\right)|Z\sim T_{j}/\left(\left|A_{j}\right|-\left|B_{j}\right|\right)\sim F_{\left|A_{j}\right|-\left|B_{j}\right|,n-\left|A_{j}\right|};\;1\leq j\leq p,$$

*where F*_*d*_1_,*d*_2__ *denotes the F-distribution with d*_1_ *and d*_2_ *degrees of freedom*.

Proof of Lemma 11

Let **Z** = (**Y**, **X**) as in (13). Given data submatrix $Z_{A_{j}}$,

$$\frac{e_{j}^{⊤}\left(P_{A_{j}}-P_{B_{j}}\right)e_{j}}{\sigma_{j}^{2}}\left|Z_{A_{j}}\sim \chi_{\left|A_{j}\right|-\left|B_{j}\right|}^{2},\;\frac{e_{j}^{⊤}\left(I-P_{A_{j}}\right)e_{j}}{\sigma_{j}^{2}}\right|Z_{A_{j}}\sim \chi_{n-\left|A_{j}\right|}^{2},$$

and they are independent, because $P_{A_{j}}-P_{B_{j}}$ and $I-P_{A_{j}}$ are orthonormal projection matrices, $e_{j}|Z_{A_{j}}\sim N\left(0,\sigma_{j}^{2}I_{n}\right)$, and $\left(P_{A_{j}}-P_{B_{j}}\right)\left(I-P_{A_{j}}\right)=0$. Then, for any real number *t*, the characteristic function $t\mapsto 𝔼\exp\left(\iota tT_{j}/\left(\left|A_{j}\right|-\left|B_{j}\right|\right)\right)$ is (*ι* is the imaginary unit)

$$𝔼\left(𝔼\left(\exp\left(\iota tT_{j}/\left(\left|A_{j}\right|-\left|B_{j}\right|\right)\right)|Z_{A_{j}}\right)\right)=𝔼\psi_{\left|A_{j}\right|-\left|B_{j}\right|,n-\left|A_{j}\right|}(t)=\psi_{\left|A_{j}\right|-\left|B_{j}\right|,n-\left|A_{j}\right|}(t),$$

where $\psi_{\left|A_{j}\right|-\left|B_{j}\right|,n-\left|A_{j}\right|}$ is the characteristic function of F-distribution with degrees of freedom $\left|A_{j}\right|-\left|B_{j}\right|,n-\left|A_{j}\right|$. Hence, $T_{j}/\left(\left|A_{j}\right|-\left|B_{j}\right|\right)\sim F_{\left|A_{j}\right|-\left|B_{j}\right|,n-\left|A_{j}\right|}$. Similarly, we also have $T_{j}^{*}/\left(\left|A_{j}\right|-\left|B_{j}\right|\right)\sim F_{\left|A_{j}\right|-\left|B_{j}\right|,n-\left|A_{j}\right|}$; *j* = 1, … , *p*.

Next, we prove independence for ***T*** and ***T**** via a peeling argument. Let ***t*** = (*t*_1_, … , *t_p_*). Let *Y_j_* be a leaf node of the graph 𝒢. Then $T_{-j}|{\text{Y}}_{-j}$, **X** is deterministic, where ***T****_−j_* is the subvector of ***T*** with the *j*th component removed. The characteristic function of ***t***^⊤^***T*** is

(32)
$$𝔼\exp\left(\iota t^{⊤}T\right)=𝔼\left(𝔼\left(\exp\left(\iota t_{j}T_{j}\right)|Y_{-j},X\right)\exp\left(\iota t_{-j}^{⊤}T_{-j}\right)\right)\;\;\;\;\;\;\;=\psi_{\left|A_{j}\right|-\left|B_{j}\right|,n-\left|A_{j}\right|}\left(t_{j}\right)𝔼\exp\left(\iota t_{-j}^{⊤}T_{-j}\right),$$

where $\psi_{\left|A_{j}\right|-\left|B_{j}\right|,n-\left|A_{j}\right|}$ is the characteristic function of the F-distribution with degrees of freedom $\left(\left|A_{j}\right|-\left|B_{j}\right|,n-\left|A_{j}\right|\right)$. Next, let $Y_{j^{\prime }}$ be a leaf node of the graph ${𝒢}^{\prime }$ and apply the law of iterated expectation again, where ${𝒢}^{\prime }$ is the subgraph of 𝒢 without node *Y_j_*. Repeat this procedure and after *p* steps $𝔼\exp\left(\iota t^{⊤}T\right)=\prod_{j=1}^{p}\psi_{\left|A_{j}\right|-\left|B_{j}\right|,n-\left|A_{j}\right|}\left(t_{j}\right)$, which implies (*T*_1_, … , *T_p_*) are independent. Similarly, ***T**** also has independent components given **Z** and has the same distribution as ***T***. This completes the proof.

### B.5 Proof of Theorem 5

Let Lr(𝒮,Σ) denote the likelihood ratio given 𝒮 and **Σ**. Then $\mathrm{Lr}=\mathrm{Lr}(\hat{𝒮},\hat{\Sigma })$.

Proof of (A)

Without loss of generality, assume *M* is sufficiently large. For any real number *x*,

(33)
$$\;|\mathbb{P}(\mathrm{Lr}\leq x)-\mathbb{P}(\mathrm{Lr}(𝒮,\hat{\Sigma })\leq x)|\;\leq 𝔼|\text{I}(\mathrm{Lr}\leq x)-\text{I}(\mathrm{Lr}(𝒮,\hat{\Sigma })\leq x)|\;=𝔼|\text{I}(\mathrm{Lr}\leq x,\hat{𝒮}\neq 𝒮)+\text{I}(\mathrm{Lr}(𝒮,\hat{\Sigma })\leq x)(\text{I}(\hat{𝒮}=𝒮)-1)|\;\leq 2\mathbb{P}(\hat{𝒮}\neq 𝒮).$$

From (14), ${\mathrm{Lr}}^{*}\left(𝒮,{\hat{\Sigma }}^{*}\right)=\sum_{\left\{j:D_{𝒮}(j)\neq \emptyset \right\}}T_{j}^{*}$. By Lemma 11,

$$\mathbb{P}(\mathrm{Lr}(𝒮,\hat{\Sigma })\leq x)=\mathbb{P}\left({\mathrm{Lr}}^{*}\left(𝒮,{\hat{\Sigma }}^{*}\right)\leq x|Z\right).$$

Note that ${\mathrm{Lr}}^{*}={\mathrm{Lr}}^{*}\left({\hat{𝒮}}^{*},{\hat{\Sigma }}^{*}\right)$. Then for any real number *x*,

(34)
$$\;\left|\mathbb{P}\left({\mathrm{Lr}}^{*}\leq x|Z\right)-\mathbb{P}(\mathrm{Lr}(𝒮,\hat{\Sigma })\leq x)\right|\;=\left|\mathbb{P}\left({\mathrm{Lr}}^{*}\leq x,{\hat{𝒮}}^{*}\neq 𝒮|Z\right)+\mathbb{P}\left({\mathrm{Lr}}^{*}\leq x,{\hat{𝒮}}^{*}=𝒮|Z\right)-\mathbb{P}\left({\mathrm{Lr}}^{*}\left(𝒮,{\hat{\Sigma }}^{*}\right)\leq x|Z\right)\right|\;\leq \left|\mathbb{P}\left({\mathrm{Lr}}^{*}\leq x,{\hat{𝒮}}^{*}\neq 𝒮|Z\right)\right|+\left|\mathbb{P}\left({\mathrm{Lr}}^{*}\left(𝒮,{\hat{\Sigma }}^{*}\right)\leq x,{\hat{𝒮}}^{*}\neq 𝒮|Z\right)\right|\leq 2\mathbb{P}\left({\hat{𝒮}}^{*}\neq 𝒮|Z\right).$$

Since (33) and (34) hold uniformly in *x*, we have

$$\underset{x\in \mathbb{R}}{\sup}\left|\mathbb{P}(\mathrm{Lr}\leq x)-\mathbb{P}\left({\mathrm{Lr}}^{*}\leq x|Z\right)\right|\leq 2\mathbb{P}(\hat{𝒮}\neq 𝒮)+2\mathbb{P}\left({\hat{𝒮}}^{*}\neq 𝒮|Z\right).$$

By Theorem 4, we have $\mathbb{P}(\hat{𝒮}\neq 𝒮)\to 0$, which holds uniformly for all ***θ*** which satisfy the Assumptions 1-4. For $\mathbb{P}\left({\hat{𝒮}}^{*}\neq 𝒮|Z\right)$, the error terms of (7) in the perturbed data **Z**^*^ are rescaled with ${\text{Ω}}_{jj}^{-1}+{\hat{\text{σ}}}_{j}^{2}\leq 2{\text{Ω}}_{jj}^{-1}$. By Theorem 4, $\mathbb{P}\left({\hat{𝒮}}^{*}\neq 𝒮\right)=𝔼\mathbb{P}\left({\hat{𝒮}}^{*}\neq 𝒮|Z\right)\to 0$, which implies $\mathbb{P}\left({\hat{𝒮}}^{*}\neq 𝒮|Z\right)\overset{p}{\to }0$ as n→∞ by the Markov inequality. Consequently, $\sup_{x\in \mathbb{R}}\left|\mathbb{P}(\mathrm{Lr}\leq x)-\mathbb{P}\left({\mathrm{Lr}}^{*}\leq x|Z\right)\right|\to 0$. For |𝒟|=0, ℙ(Lr=0)→1, $\mathbb{P}\left({\mathrm{Lr}}^{*}=0|Z\right)\to 1$, and ℙ(Pval=1)→1. For |𝒟|>0, $\mathbb{P}\left({\mathrm{Lr}}^{*}\geq \mathrm{Lr}|Z\right)\to \mathrm{Unif}(0,1)$ and ℙ(Pval<α)→α. This completes the proof of (A).

Proof of (B)

Let ${\text{Pval}}_{k}=M^{-1}\sum_{m=1}^{M}\text{I}\left({\text{Lr}}_{k,m}^{*}\geq \text{Lr}\right)$, the p-value of sub-hypothesis H_0,*k*_. For |𝒟|<|ℋ|, there exists an edge $\left(i_{k},j_{k}\right)\in ℋ$ but $\left(i_{k},j_{k}\right)\notin 𝒟$. Then by (A), $\mathbb{P}\left(\text{Pval}={\text{Pval}}_{k}=1\right)\to 1$. For |𝒟|=|ℋ|, note that as *n*, M→∞,

$$\mathbb{P}\left(\mathrm{Pval}<\alpha \right)=\mathbb{P}\left({\mathrm{Pval}}_{1}<\alpha ,\ldots ,{\text{Pval}}_{\left|ℋ\right|}<\alpha \right)\leq \mathbb{P}\left({\mathrm{Pval}}_{1}<\alpha \right)\to \alpha .$$

Now, define a sequence ${\left\{U_{ℋ}^{\left(r\right)}\right\}}_{r\geq 1}$ such that $U_{\left(i_{1},j_{1}\right)}^{\left(r\right)}=0$ and $\min_{2\leq k\leq |ℋ|}\left|U_{\left(i_{k},j_{k}\right)}^{\left(r\right)}\right|\geq c>0$. Thus, ${\left\{U_{ℋ}^{\left(r\right)}\right\}}_{r\geq 1}$ satisfy *H*_0_. By Proposition 7, ${\text{Pval}}_{k}\overset{p}{\to }0$ for *k* ≥ 2 as *r* → ∞. Hence,

$$\underset{\begin{array}{c} n\to \infty \\ {\text{U}}_{ℋ}^{\left(r\right)}{\text{satisfies H}}_{0} \end{array}}{\mathrm{lim sup}}{\mathbb{P}}_{\theta^{\left(r\right)}}(\text{Pval}<\alpha )\geq \underset{r}{\sup}\underset{n\to \infty }{\lim}{\mathbb{P}}_{\theta^{\left(r\right)}}(\mathrm{Pval}<\alpha )=\alpha .$$

This completes the proof.

### B.6 Proof of Proposition 6

Since $\mathbb{P}(\hat{𝒮}=𝒮)\to 1$, it suffices to consider $\text{Lr}(𝒮,\hat{\Sigma })$. For |𝒟|=0, we have ℙ(Lr=0)→1. Now, assume |𝒟|>0. Then

$$2\text{Lr}\left(𝒮,\hat{\Sigma }\right)=\underset{R_{1}}{\underset{︸}{\sum_{\left\{j:D_{𝒮}\left(j\right)=\emptyset \right\}}\frac{e_{j}^{⊤}\left(P_{A_{j}}-P_{B_{j}}\right)e_{j}}{\sigma_{j}^{2}}}}+\underset{R_{2}}{\underset{︸}{\sum_{\left\{j:D_{𝒮}\left(j\right)=\emptyset \right\}}\left(\frac{\sigma_{j}^{2}}{{\hat{\sigma }}_{j}^{2}}-1\right)\frac{e_{j}^{⊤}\left(P_{A_{j}}-P_{B_{j}}\right)e_{j}}{\sigma_{j}^{2}}}}.$$

To derive the asymptotic distribution of *R*_1_, we apply the strategy with law of iterated expectation as in the proof of Lemma 11. Then we have ${\left\{e_{j}^{⊤}\left(P_{A_{j}}-P_{B_{j}}\right)e_{j}/\sigma_{j}^{2}\right\}}_{\left\{j:D_{𝒮}(j)\neq \emptyset \right\}}$ are independent. Therefore, $R_{1}∼\chi_{\left|𝒟\right|}^{2}$. To bound *R*_2_, we apply Lemma 1 of Laurent and Massart (2000). By Assumption 5,

$$\mathbb{P}\left(\underset{\left\{j:{\text{D}}_{𝒮}(j)\neq \emptyset \right\}}{\max}\left|\frac{{\hat{\sigma }}_{j}^{2}}{\sigma_{j}^{2}}-1\right|\geq 4\sqrt{\frac{\log|𝒟|}{(1-\rho )n}}+8\frac{\log|𝒟|}{(1-\rho )n}\right)\leq 2\exp(-\log|𝒟|).$$

Hence, $\underset{\left\{j:{\text{D}}_{𝒮}(j)\neq \emptyset \right\}}{\max}\left|\frac{\sigma_{j}^{2}}{{\hat{\sigma }}_{j}^{2}}-1\right|\leq 8\sqrt{\log(|𝒟|)/(1-\rho )n}$ with probability tending one. Thus

$$\left|R_{2}\right|\leq \left|R_{1}\right|\underset{\left\{j:{\text{D}}_{𝒮}(j)\neq \emptyset \right\}}{\max}\left|\frac{\sigma_{j}^{2}}{{\hat{\sigma }}_{j}^{2}}-1\right|\leq O_{\mathbb{P}}\left(|𝒟|\sqrt{\frac{\log|𝒟|}{n}}\right).$$

Consequently, the desired result follows.

### B.7 Proof of Proposition 7

Let $\theta^{\left(n\right)}=\left(U^{\circ }+\text{Δ},W^{\circ }\right)$. Then

$$L\left(\theta^{\left(n\right)},\Sigma \right)-L\left(\theta^{\circ },\Sigma \right)=\underset{\left\{j:\text{D}𝒮(j)\neq \emptyset \right\}}{\sum }\left(\sqrt{n}\eta_{j}^{⊤}{\text{Δ}}_{\cdot j}-\frac{1}{2}\sqrt{n}{\text{Δ}}_{\cdot j}\left(n^{-1}Y_{{\text{D}}_{𝒮}(j)}^{⊤}Y_{{\text{D}}_{𝒮}(j)}\right)\sqrt{n}{\text{Δ}}_{\cdot j}\right),$$

where $\eta_{j}=\left(n^{-1/2}Y_{{\text{D}}_{𝒮}(j)}^{⊤}e_{j},0_{\left|{\text{D}}_{𝒮}{\left(j\right)}^{c}\right|}\right)$ is a *p*-vector. It suffices to consider $\text{Lr}(𝒮,\hat{\Sigma })$, since $\mathbb{P}(\hat{S}=𝒮)\to 1$. Under null hypothesis *H*_0_, $2\text{Lr}=\sum_{\left\{j:{\text{D}}_{𝒮}(j)\neq \emptyset \right\}}T_{j}$, where by Proposition 6 we have $T_{j}=\sum_{r=1}^{\left|D_{𝒮}(j)\right|}{\left(q_{j,r}^{⊤}e_{j}\right)}^{2}+o_{\mathbb{P}}(1)$ with $P_{A_{j}}-P_{B_{j}}=\sum_{r=1}^{\left|{\text{D}}_{𝒮}(j)\right|}q_{j,r}q_{j,r}^{⊤}$. Letting $Q_{j}=\left(q_{j,1},\ldots ,q_{j,\left|D_{𝒮}(j)\right|}\right)$ be an $n\times |{\text{D}}_{𝒮}(j)|$ matrix, then

$$\left(\begin{array}{c} Q_{j}^{⊤}e_{j}\\ \eta_{j} \end{array}\right)Y_{{\text{PA}}_{𝒮}(j)},X∼N\left(0,\left(\begin{array}{cc} I_{\left|{\text{D}}_{𝒮}(j)\right|} & n^{-1/2}Q_{j}^{⊤}Y_{{\text{D}}_{𝒮}(j)}\\ n^{-1/2}Y_{{\text{D}}_{𝒮}(j)}^{⊤}Q_{j} & n^{-1}Y_{{\text{D}}_{𝒮}(j)}^{⊤}Y_{{\text{D}}_{𝒮}(j)} \end{array}\right)\right),$$

$$Q_{j}^{⊤}e_{j}|\eta_{j},Y_{{\text{PA}}_{𝒮}(j)},X∼N\left(n^{-1/2}Y_{{\text{D}}_{𝒮}(j)}^{⊤}Q_{j}\eta_{j},Y_{{\text{D}}_{𝒮}(j)}^{⊤}\left(I_{n}-Q_{j}Q_{j}^{⊤}\right)Y_{{\text{D}}_{𝒮}(j)}\right).$$

Next, let *Y_k_* be a leaf node of the graph 𝒢. For fixed |𝒟|>0, after change of measure,

$$\beta \left(\theta^{\circ },\text{Δ}\right)\;\geq \underset{n\to \infty }{\mathrm{lim inf}}\;{𝔼}_{\theta^{\circ }}\left(\text{I}\left(\overset{p}{\underset{j=1}{\sum }}{\left\|Q^{⊤}e_{j}\right\|}_{2}^{2}>\chi_{|𝒟|,1-\alpha }^{2}\right)\exp\left(L\left(\theta^{\left(n\right)},\Sigma \right)-L\left(\theta^{\circ },\Sigma \right)\right)\right)\;=\underset{n\to \infty }{\mathrm{lim inf}}\;{𝔼}_{\theta^{\circ }}𝔼\left(\text{I}\left(\overset{p}{\underset{j=1}{\sum }}{\left\|Q^{⊤}e_{j}\right\|}_{2}^{2}>\chi_{|𝒟|,1-\alpha }^{2}\right)\exp\left(L\left(\theta^{\left(n\right)},\Sigma \right)-L\left(\theta^{\circ },\Sigma \right)\right)|Y_{-k},X\right)\;=\underset{n\to \infty }{\mathrm{lim inf}}\;𝔼\left(\mathbb{P}\left({\left\|Z_{k}+Q_{k}^{⊤}Y_{{\text{D}}_{𝒮}(k)}{\text{Δ}}_{\cdot k}\right\|}_{2}^{2}+\underset{j\neq k}{\sum }{\left\|Q_{j}^{⊤}e_{j}\right\|}_{2}^{2}>\chi_{|𝒟|,1-\alpha }^{2}|Y_{-k},X\right)\right),$$

where $Z_{k}∼N\left(0,I_{\left|{\text{D}}_{𝒮}(k)\right|}\right)$. By Lemma 3 of Li et al. (2020), ${\left\|Q_{k}^{⊤}Y_{{\text{D}}_{𝒮}(k)}{\text{Δ}}_{\cdot k}\right\|}_{2}^{2}\geq c_{3}{\left\|{\text{Δ}}_{\cdot k}\right\|}_{2}^{2}$ with probability tending to one. Therefore, a peeling argument yields

$$\beta \left(\theta^{\circ },\text{Δ}\right)\geq \mathbb{P}\left({\left\|Z+c_{3}\text{Δ}\right\|}_{2}^{2}\geq \chi_{|𝒟|,1-\alpha }^{2}\right),$$

where $Z∼N\left(0,I_{\left|𝒟\right|}\right)$. Similarly, as |𝒟|→∞,

$$\beta \left(\theta^{\circ },H\right)\geq \mathbb{P}\left({\left\|Z+c_{3}\text{Δ}\right\|}_{2}^{2}>\sqrt{2|𝒟|}z_{1-\alpha }+|𝒟|\right)\to \mathbb{P}\left(Z>z_{1-\alpha }-c_{3}∥\text{Δ}{∥}_{2}^{2}/\sqrt{2|𝒟|}\right).$$

This completes the proof.

### B.8 Proof of Proposition 8

By Proposition 7, for fixed |𝒟|=|ℋ|>0, $\beta \left(\theta^{\circ },\text{Δ}\right)$ equals to

$$\mathbb{P}\left({\text{Pval}}_{1}<\alpha ,\ldots ,{\text{Pval}}_{\left|ℋ\right|}<\alpha \right)\geq 1-\overset{\left|ℋ\right|}{\underset{k=1}{\sum }}\mathbb{P}\left({\text{Pval}}_{k}\leq \alpha \right)\geq 1-|ℋ|\mathbb{P}\left(Z_{1}^{2}\leq \chi_{1,1-\alpha }^{2}\right),$$

where $Z_{1}∼N\left(\delta /\underset{1\leq j\leq p}{\max}{\text{Ω}}_{jj},1\right)$. Then $\mathbb{P}\left({\tilde{Z}}^{2}\leq x\right)\leq \mathbb{P}(\tilde{Z}\leq -\mu +\sqrt{x})\leq \frac{1}{\sqrt{2\pi }}\frac{e^{-{\left(\mu -\sqrt{x}\right)}^{2}/2}}{\mu -\sqrt{x}}$ for $\tilde{Z}∼N(\mu ,1)$ and any $0\leq x<\mu^{2}$ and *μ* > 0 (Small, 2010). Hence, as |𝒟|=|ℋ|→∞,

$$\beta \left(\theta^{\circ },\text{Δ}\right)\geq 1-|ℋ|\mathbb{P}\left(Z_{1}^{2}\leq \chi_{1,1-\alpha }^{2}\right)\;\;\;\;\;\geq 1-\frac{\left|ℋ\right|}{\sqrt{2\pi }}e^{-{\left(\delta \sqrt{\log|ℋ|}/\max_{1\leq j\leq p}{\text{Ω}}_{jj}-\sqrt{\chi_{1,1-\alpha }^{2}}\right)}^{2}/2}\to 1.$$

This completes the proof.

### B.9 Proof of Theorem 9

The proof proceeds in two steps to show that

- $\left\{l:\left|{\text{V}}_{lj}\right|\geq \tau_{j}^{*}\right\}\subseteq \left\{l:\hat{V_{lj}}\neq 0\right\}\subseteq \left\{l:{\text{V}}_{lj}\neq 0\right\}$ for 1≤j≤p almost surely, and
- ${\hat{𝒢}}_{+}={𝒢}_{+}$ when $\hat{V}$ satisfies the property in (A).

Proof of (A)

This proof is similar to that of Theorem 4 part (A). To proceed, let $A_{j}^{\circ }=\left\{l:{\text{V}}_{lj}\neq 0\right\}$, $A_{j}^{*}=\left\{l:\left|{\text{V}}_{lj}\right|>\tau_{j}^{*}\right\}$, and $A_{j}^{\left[t\right]}=\left\{l:\left|\hat{V_{lj}^{\left[t\right]}}\right|>\tau_{j}\right\}$. Then we define the false negative set ${\text{FN}}_{j}^{\left[t\right]}=A_{j}^{*}\backslash A_{j}^{\left[t\right]}$, and the false positive set ${\text{FP}}_{j}^{\left[t\right]}=A_{j}^{\left[t\right]}\backslash A_{j}^{\circ }$ for t≥0. Consider

$${ℰ}_{j}=\left\{{\left\|X^{⊤}{\hat{\xi }}_{j}/n\right\|}_{\infty }\leq 0.5\gamma_{j}\tau_{j}\right\}\cap \left\{{\left\|{\hat{V}}_{\cdot j}^{\circ }-V_{\cdot j}\right\|}_{\infty }\leq 0.5\tau_{j}\right\},$$

where ${\hat{\xi }}_{j}=Y_{j}-X{\hat{V}}_{\cdot j}^{\circ }$ for 1≤j≤p. Again, we shall show that ${\text{FN}}_{j}^{\left[t\right]}$, and ${\text{FP}}_{j}^{\left[t\right]}$, are eventually empty on event ${ℰ}_{j}$, which has a probability tending to one.

Assume $\left|A_{j}^{\circ }\cup A_{j}^{\left[t-1\right]}\right|\leq 2\kappa_{\max}^{\circ }$. Then (28), (29), and (30) remain true on ${ℰ}_{j}$. As a result, $\left|A_{j}^{\circ }\cup A_{j}^{\left[t\right]}\right|\leq 2\kappa_{\max}^{\circ }$ on ${ℰ}_{j}$, for t≥1.

To estimate the number of iterations required for termination, it suffices to prove that $\left|{\text{FN}}_{j}^{\left[t\right]}\right|+\left|{\text{FP}}_{j}^{\left[t\right]}\right|<1$ eventually. Suppose $\left|{\text{FN}}_{j}^{\left[t\right]}\right|+\left|{\text{FP}}_{j}^{\left[t\right]}\right|\geq 1$. For any $l\in {\text{FN}}_{j}^{\left[t\right]}\cup {\text{FP}}_{j}^{\left[t\right]}$, by Assumption 5,

$$\left|{\tilde{V}}_{lj}^{\left[t\right]}-\hat{{\text{V}}_{lj}^{\circ }}\right|\geq \left|{\tilde{V}}_{lj}^{\left[t\right]}-V_{lj}\right|-\left|\hat{{\text{V}}_{lj}^{\circ }}-{\text{V}}_{lj}\right|\geq \min\left(\tau_{j}^{*}-\tau_{j},\tau_{j}\right),$$

so $\sqrt{\left|{\text{FN}}_{j}^{\left[t\right]}\right|+\left|{\text{FP}}_{j}^{\left[t\right]}\right|}\leq {\left\|{\tilde{V}}_{\cdot j}^{\left[t\right]}-{\hat{V}}_{\cdot j}^{\circ }\right\|}_{2}/\min\left(\tau_{j}^{*}-\tau_{j},\tau_{j}\right)$. Letting $\kappa_{j}^{*}=\left|A_{j}^{*}\right|$, by (30) and the Cauchy-Schwarz inequality,

$$c_{1}{\left\|{\tilde{V}}_{\cdot j}^{\left[t\right]}-{\hat{V}}_{\cdot j}^{\circ }\right\|}_{2}\leq 1.5\gamma_{j}\tau_{j}\sqrt{\left|A_{j}^{\circ }\text{Δ}A_{j}^{\left[t-1\right]}\right|}\leq 1.5\gamma_{j}\tau_{j}\sqrt{\left|{\text{FN}}_{j}^{\left[t-1\right]}\right|+\left|{\text{FP}}_{j}^{\left[t-1\right]}\right|+\left(\kappa_{j}^{\circ }-\kappa_{j}^{*}\right)}.$$

By the conditions for (*τ_j_, γ_j_*) in Theorem 9, we have

$$\sqrt{\left|{\text{FN}}_{j}^{\left[t\right]}\right|+\left|{\text{FP}}_{j}^{\left[t\right]}\right|}\leq \frac{{\left\|{\tilde{\text{V}}}_{\cdot j}^{\left[t\right]}-{\hat{\text{V}}}_{\cdot j}^{\circ }\right\|}_{2}}{\min\left(\tau_{j}^{*}-\tau_{j},\tau_{j}\right)}\;\;\;\;\;\;\;\;\leq \frac{1.5\gamma_{j}\tau_{j}}{c_{1}\min\left(\tau_{j}^{*}-\tau_{j},\tau_{j}\right)}\left(\sqrt{\left|{\text{FN}}_{j}^{\left[t-1\right]}\right|+\left|{\text{FP}}_{j}^{\left[t-1\right]}\right|}+\sqrt{\kappa_{j}^{\circ }-\kappa_{j}^{*}}\right)\;\;\;\;\;\;\;\;\leq 0.5\sqrt{\left|{\text{FN}}_{j}^{\left[t-1\right]}\right|+\left|{\text{FP}}_{j}^{\left[t-1\right]}\right|}+0.25.$$

Hence, $\sqrt{\left|{\text{FN}}_{j}^{\left[t\right]}\right|+\left|{\text{FP}}_{j}^{\left[t\right]}\right|}\leq {\left(1/2\right)}^{t}\sqrt{\left|A_{j}^{\circ }\right|+\left|A_{j}^{\left[0\right]}\right|}+0.5.$. For $t\geq 1+\left\lceil \log\kappa_{j}^{\circ }/\log4\right\rceil$, we have $\left|{\text{FN}}_{j}^{\left[t\right]}\right|+\left|{\text{FP}}_{j}^{\left[t\right]}\right|<1$ implying that ${\text{FN}}_{j}^{\left[t\right]}=\emptyset$ and ${\text{FP}}_{j}^{\left[t\right]}=\emptyset$.

Under the conditions for (*τ_j_, γ_j_*) in Theorem 9, using the same argument in Proof of Theorem 4 we have

$$\mathbb{P}\left(\left\{l:\left|{\text{V}}_{lj}\right|>\tau_{j}^{*}\right\}\subseteq \left\{l:\left|{\tilde{V}}_{lj}\right|>\tau_{j}\right\}\subseteq \left\{l:{\text{V}}_{lj}\neq 0\right\};1\leq j\leq p\right)\geq 1-\sqrt{\frac{2}{\pi }}pq^{-3}n^{-4}.$$

By Borel-Cantelli lemma, almost surely we have

$$\left\{l:\left|{\text{V}}_{lj}\right|>\tau_{j}^{*}\right\}\subseteq \left\{l:\left|{\tilde{V}}_{lj}\right|>\tau_{j}\right\}\subseteq \left\{l:{\text{V}}_{lj}\neq 0\right\},\;j=1,\ldots ,p,$$

when *n* is sufficiently large. Thus, we have $\left\{l:\hat{{\text{V}}_{lj}}\neq 0\right\}=\left\{l:\left|{\tilde{\text{V}}}_{lj}\right|>\tau_{j}\right\}$ whenever $\kappa_{j}=\left|\left\{l:{\tilde{\text{V}}}_{lj}>\tau_{j}^{*}\right\}\right|$; *j* = 1, … , *p*. The desired result follows.

Proof of (B)

This is the same as the Proof of Theorem 4 part (B).

### B.10 Proof of Theorem 10

By Theorem 4, it suffices to consider the event $\left\{{\hat{𝒢}}_{+}={𝒢}_{+}\right\}$. Then with **X** being replaced by $\left(Y_{\cdot ,\text{AN}{𝒢}_{+}\left(j\right)},X_{\cdot ,\text{IN}{𝒢}_{+}\left(j\right)}\right)$, this proof is almost the same as that of Theorem 4 part (A) and thus is omitted.

## Appendix C. Supplements for simulations

### C.1 Implementation details

DP-LR and LR:

- For Algorithm 3, we fix the Monte Carlo sample size *M* = 500. Our limited numerical experience suggests that this choice appears adequate for our purpose.
- For Algorithms 1 and 2, we choose $\tau_{j}\in \{0.05,0.1,0.15\}$ and $\gamma_{j}=\exp\left(\gamma_{j}^{\prime }\right)$ with
  $$\gamma_{j}^{\prime }\in \left\{\log\left(\underset{l,j}{\max}\left|X_{\cdot l}^{⊤}Y_{\cdot j}\right|\right),\ldots ,0.05\log\left(\underset{l,j}{\max}\left|X_{\cdot l}^{⊤}Y_{\cdot j}\right|\right)\right\}\;(\text{100 equally spaced values}).$$
  Then BIC is used to estimate tuning parameters $\kappa_{j}\in \{1,\ldots ,30\}$ ; *j* = 1, … , *p*.

2SPLS (Chen et al., 2018):

- We use the R package BigSEM for 2SPLS. In our experiments, the *λ* sequence of 2SPLS (Chen et al., 2018) is set in the same way as the *γ* sequence of the proposed methods. We use the default settings for other parameters.

### C.2 Additional simulations

This section supplements Section 5.2 by including additional numerical experiments.

#### C.2.1 Random graphs with different sparsity

We examine the proposed method for structure learning of DAGs with different sparsity. For **U**, the upper off-diagonal entries U_*kj*_; *k* < *j* are sampled independently from {0, 1} according to Bernoulli(*s/p*); *s* = 1, 2, 3, 4, while other entries are zero. The rest of the settings remain the same as in Section 5.2. Figure 11 displays the results. As expected, the performance improves when the sample size *n* increases and the DAG becomes sparser (controlled by *s*).

#### C.2.2 SHD transition curves of structure learning

We consider different sample sizes *n* = 50, 100, 150, 200 to further examine how the proposed method depends on *n*. Figure 12 displays the SHD transition curves of the peeling algorithm.

#### C.2.3 Structure learning with different numbers of interventions

Finally, we investigate how the number of interventions *q* affects the learning outcomes. Figure 13 displays the results when *q* = 500, 1000, 1500. It suggests that the proposed method performs reasonably well at a moderate sample size when many unknown interventions are present.
